# Supplementary material for: Assessing the role of children in the COVID-19 pandemic in Belgium using perturbation analysis
Source: Nat Commun. 2025 Mar 5;16:2230. doi: 10.1038/s41467-025-57087-z (PMC11882900; doi:10.1038/s41467-025-57087-z)
Supplement: Supplementary file 1 — Supplementary Information [file 41467_2025_57087_MOESM1_ESM.pdf]

Supplementary Information

Assessing the Role of Children in the COVID-19 Pandemic in Belgium Using Perturbation Analysis

Leonardo Angeli[1,*], Constantino Pereira Caetano[2], Nicolas Franco[1,3], Pietro Coletti[1], Christel Faes[1], Geert Molenberghs[1,4], Philippe Beutels[5], Steven Abrams[1,6], Lander Willem[5,6], and Niel Hens[1,5]

[1]Data Science Institute, I-BioStat, Hasselt University, Hasselt, Belgium
[2]Center for Computational and Stochastic Mathematics, Instituto Superior Técnico, University of Lisbon, Portugal
[3]Namur Institute for Complex Systems (naXys) and Department of Mathematics, University of Namur, Namur, Belgium
[4]L-BioStat, KU Leuven, Leuven, Belgium
[5]Centre for Health Economics Research and Modelling Infectious Diseases, Vaccine & Infectious Disease Institute, University of Antwerp, Antwerp, Belgium
[6]Department of Family Medicine and Population Health (FAMPOP), University of Antwerp, Antwerp, Belgium
[*]leonardo.angeli@uhasselt.be

Supplementary Methods

In infectious disease modelling, understanding the potential for an outbreak to grow or decline is paramount. One popular approach to address the issue and quantify the above potential is the basic reproduction number, denoted as R_0, which represents the average number of secondary infections produced by an infectious individual in an entirely susceptible population. Central to this notion is the next generation matrix (NGM or $\mathbf{K}$), which serves as a fundamental tool to derive this crucial metric and acts as an operator projecting one generation of infected individuals onto the subsequent, when disease dynamic is conceptualized in terms of sequential generational steps [4, 12]. For models accounting for heterogeneity - whether due to age, location, or other classifications - the NGM entry k_{ij} denotes the average number of secondary cases of type i caused by a primary case of type j during its infectious period[11, 16]. The dominant eigenvalue of this matrix gives R_0. To define the structure of our model, we specify the 'type' referenced above as corresponding to discrete age groups into which the population is divided. In age-structured models, performing perturbation (or formal sensitivity) analysis on the NGM can elucidate intricate interactions and dependencies between different age groups and virus transmission, providing insights for targeting intervention strategies. The NGM connects the model structure, epidemiological parameters, and the potential for disease spread.

Adopting the methodology delineated by Diekmann et al.[12], we extract the next-generation matrix from the compartmental model presented in the research of Abrams et al.[1]. Subsequently, building on the methodology defined by Angeli et al.[4], we conduct a formal perturbation analysis of the aforementioned NGM, which leads to the identification of sensitivity indices. These indices enable us to unravel the evolving role of different age groups in the virus transmission dynamics within Belgium.

We briefly recall that, in our case, the NGM (or $\mathbf{K}$) is a matrix with exclusively positive entries, signifying its spectral radius $\rho(\mathbf{K})$ is a strictly positive and algebraically simple eigenvalue of the NGM [22]. Additionally, this dominant (or Perron) eigenvalue is associated with real-valued right ($\mathbf{w}$) and left ($\mathbf{v}$), the only eigenvectors of $\mathbf{K}$ whose components are all strictly positive. As previously noted, $\mathbf{K}$ dominant eigenvalue is interpreted as the basic reproduction number R_0 for the disease under study, assuming the population is fully susceptible (a reasonable assumption during the initial stages of disease spread). Nonetheless, as the epidemic progresses, vaccination strategies are rolled out, and virus strains diversify, this assumption is no longer valid, as a proportion of the population will develop some immunity against infection. Provided we can estimate this proportion for n age classes ($\mathbf{S} \in \mathbb{R}^n$), we define the linear operator $\mathbf{K}(\mathbf{S}) := \mathbf{K}_\mathbf{S}$, whose spectral radius offers an approximation of the effective reproduction number

(R_t), i.e., the mean number of secondary infections generated by a single infectious individual within a population where full susceptibility is not a given, see paragraph "Susceptible individuals" below. Given that our observation begins when the pandemic has already progressed, altering population susceptibility, we will primarily work with the next generation matrix $\mathbf{K}(\mathbf{S})$, adjusted for the susceptibility levels across different age groups. For the sake of simplicity in notation and to enhance readability, we will continue to refer to this adjusted matrix simply as $\mathbf{K}$. Regarding the eigenvectors associated with R_0 (or R_t), after rescaling them such that $\|\mathbf{w}\| = 1$ and $< \mathbf{w}, \mathbf{v} >= 1$ [6], $\mathbf{w}$ can be interpreted as the vector enclosing the relative disease incidence in each age group[16], while $\mathbf{v}$ as the vector enclosing age-specific per susceptible contributions to the number of new infections at each generation[4]. Finally, exploiting the definition of right and left eigenvector[5], we can derive a fundamental result, that is:

$$s_{ij} := \frac{\partial R_t}{\partial k_{ij}} = \frac{v_i w_j}{< \mathbf{w}, \mathbf{v} >} = v_i w_j, \tag{1}$$

where the subscripts i and j indicate the age groups. In (1) and in the definitions of the indices below, derivatives are understood to be evaluated at a fixed point in time. To streamline the notation, we will omit the double subscript used in the main text for the cumulative indices (denoting age group and time point), retaining the subscript j indicating the age groups.

Cumulative sensitivities

Mathematically, the cumulative sensitivity indices are defined as the Manhattan norm of the gradient of R_t w.r.t the column elements corresponding to a specific age group j. We indicate with $\mathbf{k}_j$ the j-th column of $\mathbf{K}$. The cumulative sensitivities [4] are defined as:

$$\tilde{s}_j := \left\| \nabla R_{t(\mathbf{k}_j)} \right\|_1 = \sum_{i=1}^{n} s_{ij} = w_j \left\| \mathbf{v} \right\|_1. \tag{2}$$

The index $\tilde{s}_j$ approximates the rate of change in R_t when any or some of the epidemiological parameters relative to age group j are perturbed, generating a shift in the expected number of secondary infections caused by an index case in age group j during its infectious period, i.e. in $\mathbf{k}_j$. Higher values of $\tilde{s}_j$ identify a higher potential impact on R_t of a single infected in age group j. Equation (2) also tells us that the linear change in R_t imposed by epidemiological variations in age group j is proportional to the relative incidence in that group (w_j). This is true provided no significant perturbations of the epidemiological landscape occur [4]. In our study, age groups are classified based on sensitivity indices derived from a longitudinal observation, enabling categorization by their effect on transmission dynamics. In the case of the index $\tilde{s}_j$, its average value over the observation period is assessed at $\tilde{s}_{avg} = 1(0.98, 1.03)$, 99% CI. To give an interpretation of this average sensitivity value, consider a scenario where implementing mandatory mask-wearing is expected to reduce secondary infections from an infected individual by Δ. Each k_{ij} element in the next-generation matrix $\mathbf{K}$ represents the expected number of secondary infections in group i from an infected individual in group j during their infectious period. Hence, the sum of the j-th column elements corresponds to the number of secondary infections an index case in age group j is expected to generate while infectious[4, 11]. A uniform reduction in transmission across all age groups due to mask-wearing in group j implies $\Delta = \delta(\sum_{i=1}^{n} k_{ij}) = ndk_j$. Here, n is the number of age groups, and $dk_j = \frac{\Delta}{n}$ represents a uniform variation in $\mathbf{K}$'s j-th column elements. The change in R_t due to mask-wearing in age group j is approximated using a first-order Taylor expansion:

$$\delta R_t \approx \sum_{i=1}^{n} dk_{ij} \frac{\partial R_t}{\partial kij} = \frac{\Delta}{n} \times \tilde{s}_j,$$

where the last equality holds using (2). Thus, if $\tilde{s}_j = 1$, a change Δ in the number of infections by an individual of age j impacts R_t by the quantity $\frac{\Delta}{n}$. If the cumulative sensitivity index were 1 for all groups, the total impact on R_t would equal Δ, aligning with R_t's definition.

Infective values

We propose an alternative perspective on the left eigenvector $\mathbf{v}$ components associated with R_t. For each row i in the next-generation matrix $\mathbf{K}$, denoted as $\mathbf{k}_i^*$, we evaluate the Manhattan norm of the gradient

of R_t w.r.t it, leading to:

$$\left\|\nabla R_{t(\mathbf{k}_i^*)}\right\|_1 = \sum_{j=1}^{n} s_{ij} = v_i \left\|\mathbf{w}\right\|_1 = v_i. \tag{3}$$

Here, v_i approximates the rate of change in R_t due to perturbations in epidemiological parameters that influence either the force of infection exerted on individuals in age group j or their susceptibility[4]. This change would reflect in the i-th column of the next-generation matrix, $\mathbf{k}_i^*$. Higher v_i values indicate that a single new infection in age group i will significantly impact the long-term infection count and subsequently R_t. In other terms, the impact of a single new infection in age i on future infection generations is proportional to v_i. Analogous to the concept of reproductive value in population dynamics[5], the infective value, v_i, represents the potential of an age group to perpetuate infection spread across generations. This interpretation hinges on the ergodic property of the discrete dynamical system with $\mathbf{K}$ as its projection matrix[4, 5]. The vector of new infections across the various age groups at generation m ($\mathbf{I}^{(m)}$) asymptotically converges to $R_t^m C\mathbf{w}$, where C is a constant depending on the initial distribution of infections and the infective values vector $\mathbf{v}$. Here, $\mathbf{w}$ is the right eigenvector of $\mathbf{K}$ corresponding to R_t. A single infection in age group j will increase new infections at generation m by $R_t^m \cdot v_j$[4]. We observe that an infective value $v_j = 1$ implies that infections in age group j propagate as a geometric series with a rate of R_t, reflecting the average reproduction rate of the infected population. However, we get a slightly but significantly lower average for the infective value index, evaluated at $v_{avg} = 0.93(0.90, 0.95)$, 99% CI.

Cumulative elasticities

Considering the value of the R_t and each k_{ij} leads to defining the proportional response of R_t to a proportional change in a specific column of $\mathbf{K}$. Namely:

$$\tilde{e}_j := \left\|\frac{\mathbf{k_j}}{R_t} \circ \nabla R_{t(\mathbf{k}_j)}\right\|_1 = \sum_{i=1}^{n} \frac{k_{ij}}{R_t} s_{ij} = \sum_{i=1}^{n} e_{ij}. \tag{4}$$

Given the property that R_t—when treated as a function of the entries of $\mathbf{K}$—is homogeneous of degree 1 [11], it follows that $\sum_{j=1}^{n} \tilde{e}_j = 1$. These indices can be interpreted as the per-generation average proportional contribution to R_t of age group j.

Composed Sensitivities

To analyze R_t variations more precisely, one can differentiate it w.r.t epidemiological rates that constitute k_{ij} (see equation (16) below). These lower-level parameters, denoted as l, yield sensitivities through the chain rule:

$$s_l = \frac{\partial R_t}{\partial l} = \sum_{i,j} s_{ij} \frac{\partial k_{ij}}{\partial l}. \tag{5}$$

These sensitivities are crucial for assessing the impact of specific epidemiological parameter changes on R_t.

Prospective indications

By stopping at the first order the Taylor expansion of R_t as a function of $\mathbf{K}$'s entries, it is easy to show that for small perturbations of the said entries (δk_{ij}) the two following approximation holds[4]:

$$\delta R_t \approx \sum_{i,j} s_{ij} \delta k_{ij}, \tag{6}$$

and considering a small perturbation in the parameter l, the change in R_t is approximated by

$$\delta R_t \approx \sum_{i,j} s_{ij} \frac{\partial k_{ij}}{\partial l} \delta l. \tag{7}$$

When considering proportional variations of the entries ($\eta_{ij} = \frac{\delta k_{ij}}{k_{ij}}$), the elasticities can be used to approximate the proportional change in R_t:

$$\frac{\delta R_t}{R_t} \approx \sum_{i,j} e_{ij} \eta_{ij}. \tag{8}$$

The sensitivity of R_t to all the entries of $\mathbf{K}$, k_{ij}, is bounded from above by an overall sensitivity index[5] defined as follows:

$$|dR_t| = \left\|\mathbf{v}^T d\mathbf{K}\mathbf{w}\right\| \leq \left\|\mathbf{v}^T\right\| \left\|d\mathbf{K}\right\| \left\|\mathbf{w}\right\| = S_b \left\|d\mathbf{K}\right\| \tag{9}$$

where $\|\cdot\|$ indicates the Euclidean norm for vectors and the Frobenius norm for matrices. The index S_b is defined as

$$S_b = \left\|\mathbf{v}^T\right\| \left\|\mathbf{w}\right\| = \sqrt{\sum_{i,j} \left(\frac{\partial R_t}{\partial k_{ij}}\right)^2} \tag{10}$$

and gives an upper bound on the magnitude of the change in R_t produced by a change in the next generation matrix of magnitude $\|d\mathbf{K}\|$. At each observation point in time $t = 1, \ldots, 35$, i.e. each update of the NGM corresponding to the social contact pattern returned by wave $t + 9$ of the CoMix survey (Supplementary Table 2) and susceptibility profile, we evaluate the Frobenius norm of $\|d\mathbf{K} = \mathbf{K}(t) - \mathbf{K}(t-1)\|$ and give an upper bound for the absolute variation in R_t corresponding to the updated NGM as follows:

$$\Delta_{R_{max}} = S_b \left\|d\mathbf{K}\right\|. \tag{11}$$

In Supplementary Table 1, we report the evolution of $\Delta_{R_{max}}$ throughout our observation period.

Second-Order Derivatives

We can extend the derivation of R_t to measure how variations in the NGM entries affect the quadratic variation of R_t. For our study, the significance of second-order derivatives lies primarily in measuring the variation in sensitivity and elasticity. We focus on the variation in the contribution to R_t by a specific age group j, as quantified by cumulative elasticity $\tilde{e}_j$.

For each entry k_{lm}, we have:

$$\frac{\partial \tilde{e}_j}{\partial k_{lm}} = \frac{\partial \sum_{i=1}^{n} e_{ij}}{\partial k_{lm}} = \sum_{i=1}^{n} \frac{\partial e_{ij}}{\partial k_{lm}}$$

Assuming the NGM ($\mathbf{K}$) has n distinct eigenvalues and that the variation in k_{lm} occurs independently from any other $\mathbf{K}$'s entry, we have:

$$\frac{\partial e_{ij}}{\partial k_{lm}} = \frac{k_{ij}}{R_t} \frac{\partial^2 R_t}{\partial k_{ij} k_{lm}} - \frac{k_{ij}}{R_t^2} s_{ij} s_{lm} + \delta_{il}\delta_{jm} \frac{s_{ij}}{R_t}$$

where δ_{ij} is the Kronecker delta. Using the chain rule and eigenvalue properties, the second-order sensitivity is:

$$\frac{\partial^2 R_t}{\partial k_{ij} k_{lm}} = s_{im} \sum_{z \neq 1} \frac{s_{lj}^{(z)}}{R_t - \lambda_z} + s_{lj} \sum_{z \neq 1} \frac{s_{im}^{(z)}}{R_t - \lambda_z} \tag{12}$$

where the eigenvalues of $\mathbf{K}$ are arranged in decreasing order ($R_t = \lambda_1, \lambda_2, \ldots$), and

$$s_{im}^{(z)} := \bar{v_i}^{(z)} w_m^{(z)}.$$

Here, $\mathbf{v}^{(z)}$ and $\mathbf{w}^{(z)}$ are the left and right eigenvectors corresponding to eigenvalue λ_z, with $\bar{v_i}^{(z)}$ indicating the i-th component of the conjugate of $\mathbf{v}^{(z)}$ and $w_m^{(z)}$ the m-th component of $\mathbf{w}^{(z)}$.

To express the gradient of $\tilde{e}_j$ w.r.t the m-th column vector of $\mathbf{K}$, $\mathbf{k}_m$, we obtain:

$$\frac{\partial \tilde{e}_j}{\partial \mathbf{k}_m} = J_{\mathbf{k}_m} \text{vec}(\mathbf{K})^T \nabla_{\text{vec}(\mathbf{K})} \tilde{e}_j.$$

Here, J denotes the Jacobian matrix, and the vec operator maps the matrix into an n^2 column vector by stacking the matrix columns. Assuming independence of variations (i.e., $\frac{\partial k_{ij}}{\partial k_{lm}} = \delta_{il}\delta_{jm}$), we get:

$$\frac{\partial \tilde{e}_j}{\partial \mathbf{k}_m} = \nabla_{\mathbf{k}_m} \tilde{e}_j^T = \left(\frac{\partial \tilde{e}_j}{\partial k_{1m}}, \ldots, \frac{\partial \tilde{e}_j}{\partial k_{nm}}\right) = \left(\sum_{i=1}^{n} \frac{\partial e_{ij}}{\partial k_{1m}}, \ldots, \sum_{i=1}^{n} \frac{\partial e_{ij}}{\partial k_{nm}}\right). \tag{13}$$

Interpretation

The epidemiological meaning of the elasticity gradient in Equation (13) hinges on the interpretation of the NGM entries, k_{ij}, and its columns, $\mathbf{k}_m$. Specifically, k_{ij} represents the average number of secondary cases of age i caused by a primary case of age j during its infectious period. Consequently, each column $\mathbf{k}_m$ reflects the expected distribution of secondary infections generated by an index case in age group m across all age groups during its infectious period. The gradient of the cumulative elasticity index $\tilde{e}_j$ with respect to $\mathbf{k}_m$, denoted as $\nabla_{\mathbf{k}_m}\tilde{e}_j$, provides insights into the relationship between age-specific transmission dynamics. Each component of this gradient quantifies the linear sensitivity of the proportional contribution of age group j to R_t, in response to independent variations in the transmission routes initiated by age group m. More precisely, it allows us to assess which specific transmission routes (i.e., secondary infections from m to all other age groups, k_{im} for all i) are most likely to influence the proportional contribution of age group j to overall transmission (i.e. $\tilde{e}_j$).

By summing the components of the gradient $\nabla_{\mathbf{k}_m}\tilde{e}_j$, expressed as $\mathbf{1}^T\nabla_{\mathbf{k}_m}\tilde{e}_j$, we derive an aggregated measure of the linear response of $\tilde{e}_j$ to variations in all transmission routes associated with age group m. This sum approximates how changes in the transmission potential of age group m (i.e. in the entries of $\mathbf{k}_m$) influence the proportional contribution of age group j (i.e., $\tilde{e}_j$) to overall transmission dynamics. In the Supplementary Discussion, we demonstrate how this quantity can aid in interpreting the performed counterfactual exercises, offering insights into observed shifts in the cumulative elasticity patterns across age groups. It is important to note that this approach assumes independent variations across the NGM components, which may not always hold due to correlations among $\mathbf{K}$ entries. Nevertheless, it provides a useful heuristic for exploring the interplay between transmission dynamics across different age groups.

Overall, cumulative elasticity indices provide a measure of the proportional contribution of each age group to R_t, while its gradient quantifies how this contribution might shift in response to changes in behavioural or epidemiological parameters within one or more age groups.

NGM spectral property

Some preliminary information on whether a group is likely to be a major contributor to the epidemic outbreak can be obtained directly from the NGM by exploiting well-known properties of square matrices. Specifically, the spectral radius ρ of a square matrix $\mathbf{M}$ is defined as the maximum of the absolute value of $\mathbf{M}$'s eigenvalues, *i.e.* $\rho(\mathbf{M}) := \max_j |\lambda_j|$, and satisfies the following inequalities[22]:

$$\min_i r_i \leq \rho(\mathbf{M}) \leq \max_i r_i, \tag{14}$$

$$\min_j c_j \leq \rho(\mathbf{M}) \leq \max_j c_j. \tag{15}$$

The notations r_i and c_j represent the sum of elements in row i and column j, respectively. In our case, $\rho(\mathbf{K}) = R_t$. Hence, we can use the above inequalities to determine which age groups allow for an R_t above 1 (pandemic outbreak). The last paragraph of the Supplementary Discussion interprets the evolution of the NGM's rows (Supplementary Figure 12) and columns (Supplementary Figure 11) over the study period.

Social Contact Data

Social contact data play a critical role in shaping the next generation matrix and thus directly influencing the understanding of virus transmission dynamics throughout our analysis. The selected waves of the CoMix survey conducted in Belgium[9, 27] provide the necessary parameters to evaluate and quantify the average daily interactions between different age groups, effectively constructing the social contact matrix $\mathbf{M}$. Namely, $\mathbf{M}$'s general entry m_{ij} is informed by the average daily number of contacts made by an individual in age group i with individuals in age group j. Furthermore, we adjust the social matrices to account for inherent biases such as changes in social behaviours following symptom onset and contact under-reporting due to survey fatigue. These adjustments improve the accuracy of our models and allow for a more realistic assessment of the virus transmission potential. Behavioural changes due to the onset of symptoms can be modelled by estimating the location-specific proportional change in contacts of symptomatic cases, indicated here as $\boldsymbol{\xi} = (\xi_{home}, \xi_{work}, \xi_{school}, \xi_{transport}, \xi_{leisure}, \xi_{other})$. We obtain the matrices of symptomatic interaction rates by weighting the location-specific matrices available from the CoMix survey[9], namely

$$\mathbf{M}_{asym} = \mathbf{M}_{home} + \mathbf{M}_{work} + \mathbf{M}_{school} + \mathbf{M}_{transport} + \mathbf{M}_{leisure} + \mathbf{M}_{other}$$

$$\mathbf{M}_{sym} = \xi_{home}\mathbf{M}_{home} + \xi_{work}\mathbf{M}_{work} + \xi_{school}\mathbf{M}_{school} + \xi_{transport}\mathbf{M}_{transport} + \xi_{leisure}\mathbf{M}_{leisure} + \xi_{other}\mathbf{M}_{other}$$

Due to the lack of studies on this topic at the beginning of our work, we will assume that the change in contact rates due to symptom onset is the same as observed during the 2009 A/H1N1 pandemic influenza in England [26], namely $\boldsymbol{\xi} = (1, 0.09, 0.09, 0.13, 0.06, 0.25)$. In response to a study[19] investigating potential biases in the daily contacts reported throughout the CoMix survey, we concentrate on the impact of survey fatigue within our distinct observation period (i.e., CoMix waves 9 to 43). This study points to a steady 20% underreporting factor across all the investigated survey waves and all age groups considered. Accordingly, we employ the wave- and age-specific underreporting factors offered by the study, assuming a homogenous correction for contacts made by age groups within $[0, 18), [18, 70)$ and $[70, \infty)$ at every contact matrix update.

Compartmental Model

This analytical framework is based on a recent model [1] developed to describe the transmission dynamics of SARS-CoV-2 in Belgium. It is a Susceptible, Exposed, Infectious, Recovered (SEIR) compartmental model, from which we derive the NGM . In this model, $\mathbf{S}(t)$ represents the population susceptible to infection at time t. After effective contact between a susceptible and infectious individual, the susceptible person moves to the exposed state $\mathbf{E}(t)$ at a rate $\boldsymbol{\alpha}(t)$, known as the force of infection (with boldface indicating age-specific rates). After a latent period, the individual becomes infectious and transitions to the pre-symptomatic state $\mathbf{I}_{pre}(t)$ at a rate γ. After an average time of $\frac{1}{\theta}$ days in the pre-symptomatic compartment, individuals either develop symptoms, moving to the symptomatic state $\mathbf{I}_{mild}(t)$ with probability $1 - \mathbf{p}$, or remain asymptomatic in the compartment $\mathbf{I}_{asym}(t)$ with probability $\mathbf{p}$. Asymptomatic cases recover at a uniform rate δ_1. Mild symptomatic infections either recover at a rate $\boldsymbol{\delta}_2$, or progress to a severe state $\mathbf{I}_{sev}(t)$ at a rate $\boldsymbol{\psi}$, which corresponds to cases requiring hospitalization. Although the model includes disease-related mortality for hospitalized cases, these factors do not affect the NGM structure [4]. Thus, we assume that the recovered class $\mathbf{R}(t)$ absorbs individuals who were hospitalized, including those who died after hospitalization, under the assumption that they no longer contribute to new infections (neglecting the phenomenon of nosocomial infections observed during the pandemic). Hospitalized individuals recover or die at a rate $\boldsymbol{\omega}$ and transition to the recovered compartment. The described transitions between compartments are governed by the following system of ordinary differential equations (ODEs) (1) and are schematically represented in the diagram 2.

$$\begin{aligned}
\frac{d\mathbf{S}(t)}{dt} &= -\boldsymbol{\alpha}(t)\mathbf{S}(t), \\
\frac{d\mathbf{E}(t)}{dt} &= \boldsymbol{\alpha}(t)\mathbf{S}(t) - \gamma\mathbf{E}(t), \\
\frac{d\mathbf{I}_{pre}(t)}{dt} &= \gamma\mathbf{E}(t) - \theta\mathbf{I}_{pre}(t), \\
\frac{d\mathbf{I}_{asym}(t)}{dt} &= \theta\mathbf{p}\mathbf{I}_{pre}(t) - \delta_1\mathbf{I}_{asym}(t), \\
\frac{d\mathbf{I}_{mild}(t)}{dt} &= \theta(\mathbf{1} - \mathbf{p})\mathbf{I}_{pre}(t) - (\boldsymbol{\psi} + \boldsymbol{\delta}_2)\mathbf{I}_{mild}(t), \\
\frac{d\mathbf{I}_{sev}(t)}{dt} &= \boldsymbol{\psi}\mathbf{I}_{mild}(t) - \boldsymbol{\omega}\mathbf{I}_{sev}(t), \\
\frac{d\mathbf{R}(t)}{dt} &= \delta_1\mathbf{I}_{asym}(t) + \boldsymbol{\delta}_2\mathbf{I}_{mild}(t) + \boldsymbol{\omega}\mathbf{I}_{sev}(t).
\end{aligned}$$

Figure 1: Deterministic model equations

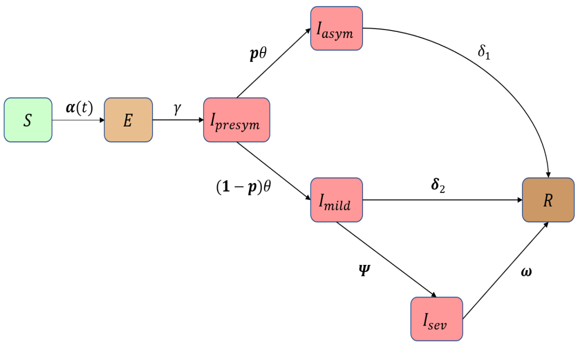

Figure 2: Model diagram

The NGM (or $\mathbf{K}$) is derived from the compartmental model by capturing the transmission dynamics as individuals progress through different epidemiological states. This derivation is presented in detail in Angeli et al.[4], and its components are given as:

$$k_{ij} = \tau_{inf} N_i \tilde{q} a_i c_{ij}^{asym} h_j \left(\frac{\delta_1 + \theta p_j}{\delta_1 \theta} \right) + N_i \tilde{q} a_i c_{ij}^{sym} h_j \frac{(\Psi_j + \omega_j)(1 - p_j)}{\omega_j (\Psi_j + \delta_{2j})}, \quad \text{for all } i, j = 1, \ldots, n, \qquad (16)$$

Here, N_i indicates the number of individuals in age group i, where the n age intervals are $[0, 6), [6, 12), [12, 18), [18, 30), [30, 40), [40, 50), [50, 60), [60, 70), [70, \infty)$. The other terms in (16) represent

model parameters, briefly described in Supplementary Table 3. In particular, a_i is the q-susceptibility of age group i (the probability of a susceptible individual of age i becoming infected after close contact), and h_j is the q-infectiousness of age group j (the probability of an infected individual of age j transmitting the virus during close contact). As in Franco et al.[14], these two quantities are defined up to a constant q, which is calibrated at each observation point so that the NGM's dominant eigenvalue matches the value of R_t estimated from positive PCR tests [15]. τ_{inf} is the infectivity ratio, i.e., the relative infectiousness of asymptomatic individuals compared to symptomatic ones, set to 0.51 based on [1]. In this framework, k_{ij} represents the expected number of infections in age group i generated by an infected individual in age group j during their infectious period. Specifically, k_{ij} can be viewed as a weighted sum of contributions from pre-symptomatic, asymptomatic, and symptomatic (mild or severe) individuals. The weights are determined by the parameters governing transitions between infection states, the time spent in these states, and the contact rates associated with each state (see paragraph "Social Contact Data" above). Further details on the model parameters, their estimates, and the analytical formulation of the NGM entries can be found in Abrams et al.[1] and Angeli et al.[4].

Assumptions

This subsection lists the assumptions for our perturbation analysis.

- **Age intervals:** we consider the following age groups (with ages in years):

$$\Omega = \{[0, 6), [6, 12), [12, 18), [18, 30), [30, 40), [40, 50), [50, 60), [60, 70), [70, \infty)\}.$$

  The age structure mimics the Belgian school system for individuals under 18 years of age. The notation Ω thus denotes the set of age intervals.

- **Contact rates**: for each survey wave, we consider the sample mean of the reported contacts as a proxy for the per capita contact rates, c_{ij} in (16). The considered waves of the CoMIx social contact survey lack data on contacts made by children under 18 years of age. Following [25], we estimated these contact rates using pre-pandemic social contact data from Flanders, Belgium. We adjusted them based on the observed relative changes in reported contact rates in other age groups (available from the CoMix study). No social contact survey data were collected between the beginning of September and the beginning of November 2020.

- **Epidemiological parameters estimates:** we assume the q-susceptibility to be age-specific, following [10], namely

$$\mathbf{a} = (a_i)_{i=1,\ldots,n} = (0.4, 0.39, 0.38, 0.79, 0.86, 0.8, 0.82, 0.88, 0.74).$$

  Assessing the age-dependence of q-infectiousness is not trivial [14, 18]). Following [14], we infer that the average infectiousness of individuals in each age group is proportional to the probability for an infected individual to remain asymptomatic $\mathbf{p}$, with $\mathbf{1}-\mathbf{p}$ being the probability of developing symptoms. For any age group j, we impose that $h_j = \tau_{inf} p_j + 1(1 - p_j)$. The resulting age-specific q-infectiousness profile is:

$$\mathbf{h} = (h_i)_{i=1,\ldots,n} = (0.54, 0.55, 0.56, 0.59, 0.7, 0.76, 0.9, 0.99, 0.99).$$

- **Reproduction number:** We choose to set $R_0 = 3.4$ (95% C.I. $(3.36, 3.44)$) for SARS-CoV-2 spread in Belgium, in agreement with [8, 28]. This corresponds, in our model, to a proportionality factor of $\tilde{q} = 0.137$, which will then be rescaled for the dominant eigenvalue of $\mathbf{K_S}$ to coincide with the R_t. The effective reproduction number (R_t) is derived as the seven-day average centred around each survey wave date, using daily mean R_t estimates from the study by Gressani et al. [15].

Susceptible individuals

In infectious disease modelling, accurately estimating the evolution of the susceptible population is crucial, especially over extended timeframes like those observed during the COVID-19 pandemic. As discussed above, the NGM is effective for assessing outbreak potential in a naive population and understanding early transmission dynamics. However, this approach relies on a linearization that overlooks changes in the susceptible population, which become significant over longer periods. Factors such as susceptible depletion due to infection, shifts in immunity from vaccination, the emergence of new virus

variants, and infection history are essential to consider to support a comprehensive longitudinal study. In the absence of comprehensive longitudinal seroprevalence studies for the Belgian population, susceptibility evolution was numerically simulated using a stochastic compartmental model developed by Willem et al. (2024)[29]. This model incorporates the circulation of various SARS-CoV-2 strains, simulating their succession as dominant variants during the observation period. The simulations are consistent with virus sequencing data from Belgium's National Reference Laboratory[17]. The model accounts for the replacement of the original strain by more transmissible Variants of Concern (VOCs), such as the Alpha, Beta, and Gamma variants, aggregated as Alpha VOC infections. Additionally, the model explicitly includes the Delta VOC, characterized by its increased transmissibility and a hospitalization hazard ratio (2.26 times that of the Alpha VOC [24]). The emergence of the Omicron VOC is also modelled, incorporating variations in transmissibility, VOC-specific latent periods, and hospitalization risks [29]. A key extension in this model is implementing a "leaky" vaccine approach, where vaccine effectiveness reduces the probability of infection and severe disease rather than providing absolute protection. The model differentiates levels of protection based on vaccine type (mRNA or adenovirus-based) and the number of doses received, including booster shots. Protection is modelled to be fully effective 21 days after the first dose, with additional doses (second and booster) reaching full effectiveness 7 days after administration. The model also addresses waning immunity, where individuals transition from full protection to a susceptible state over approximately 90 days, reflecting a gradual decrease in immunity after vaccination or infection. Importantly, the model distinguishes between various levels of protection against infection and hospitalization, depending on the vaccine type and dose, and incorporates specific adjustments for the effects of different Variants of Concern (VOCs). At each time point corresponding to a CoMix survey wave, the model tracks the number of susceptible individuals, comprising those who have never been infected or vaccinated (naive population) and individuals across four distinct categories of waning immunity. These categories are defined by the number of vaccinations received and whether the individual has previously contracted the virus. This approach ensures that our estimates accurately reflect the evolving immunity landscape, which is crucial for understanding transmission dynamics over time. Detailed levels of vaccine- and infection-induced protection, along with associated waning rates, are presented in Supplementary Table 4. A comprehensive explanation of all modelling choices is available in the study by Willem et al.[29].

Supplementary Discussion

The analysis presented in this study provides measures to quantify the impact of perturbations in biological (e.g., susceptibility and infectiousness) and behavioural (e.g., social contacts) parameters on the transmission potential of SARS-CoV-2, as measured by the effective reproduction number (R_t). Utilizing the mathematical properties inherent in our methodology, the sensitivity measures provide a hierarchy of the contribution of different age groups to virus transmission over time. These measures integrate variations in social contacts and the numerically simulated age-specific number of susceptible individuals to capture the transmission dynamics corresponding to the 81 ($n \times n$) different interactions defined in the model, ultimately influencing R_t.

However, these indices do not allow us to disentangle the specific mechanistic drivers behind the variations in age-specific transmission contributions. We briefly address this limitation empirically by exploring several counterfactual scenarios. The aim is to illustrate how specific variations in age-specific contributions to the transmission can be assessed, thereby clarifying the relative roles of changes in the susceptible population, driven by changes in the immunity and force of infection, versus changes in contact patterns.

We construct two different counterfactual NGMs for selected waves and age groups to isolate the impact of the above changes on sensitivity indices:

Scenario 1: We keep the susceptible population constant for a subset of age groups and allow the other parameters to vary according to the observed or simulated data.

Scenario 2: We keep the number of daily contacts constant for individuals in a subset of age groups and allow the other parameters to vary according to the observed or simulated data.

We then follow the evolution of the perturbation indices across the chosen time interval for both scenarios.

24/12/20-02/03/21, survey waves 12 to 17

The two panels in Supplementary Figure 3 illustrate the two hypothetical scenarios around the return to face-to-face education in January 2021. During this period, the susceptible population showed minimal fluctuation (see Figure 6 in the main text), suggesting that the contribution to transmission among the youngest age groups was primarily driven by their high level of social interactions. In Scenario 1 (Supplementary Figure 3a), assuming the contacts for individuals under 18 years old follow the observed variation while keeping their proportion of susceptibles constant, the elasticity varies in line with the observed pattern (dashed line). Conversely, in Scenario 2, maintaining the contact levels from December 2020 (wave 12) significantly reduces the role of children in transmission, resulting in an R_t consistently below 1 (Supplementary Figure 3b, red asterisks). This counterfactual exercise supports our initial guess that the observed elasticity patterns are largely driven by the structure of the social contact matrix underlying our calculations.

To explore how contact patterns and epidemiological characteristics of different age groups influence the distribution of cumulative elasticity indices for individuals under 18, we evaluated the gradient of cumulative elasticities (4) using equation (13) with respect to the NGM's column vectors ($\mathbf{k}_m$). Supplementary Figure 4 shows the sum of the component of this gradient to each column of the NGM (m subscript in (13)) for a fixed age group (j subscript in (13)), evaluated using data from survey wave 13 (January 2021). The results suggest that the transmission contribution of the $[0, 6)$ group is directly proportional to that of the $[6, 12)$ group. In contrast, it is inversely proportional to the contributions of other groups. Notably, the $[18, 30)$ group has the most significant negative impact on the cumulative elasticity of both groups. (Supplementary Figures 4a and 4b). We found that a 10% reduction in the elements of $\mathbf{k}_{m=[18,30)}$ decreases the transmission contribution of the $[18, 30)$ age group by 14% while increasing the contributions of the $[0, 6)$ and $[6, 12)$ groups by approximately 9%. Supplementary Figure 4d highlights an assortative mixing pattern[21] for these groups, particularly for the $[12, 18)$ age group, which predominantly mixes with individuals of the same age. This aligns with the higher elasticity gradient norm observed for the column $\mathbf{k}_{m=[12,18)}$ compared to other groups (Supplementary Figure 4c). The elasticity gradient reveals how age groups either counteract or support each other in their proportional contributions to R_t, irrespective of R_t's value. In this case, the proportional contributions of children in $[0, 12)$ are closely tied to those of young adults in $[18, 30)$, while the $[12, 18)$ group's contribution to transmission is poorly sensitive to changes in transmission from infectious individuals in other age groups.

18/03/21-12/06/21, survey waves 18 to 24

We examine the scenarios described above for the period from late March 2021, coinciding with stricter NPIs during the Easter holidays in Belgium (lasting until late April 2021), through late June 2021, which includes a notable decrease in the susceptible population among individuals aged over 50 years, preceding the rebound in susceptibility observed in July 2021 (see Figure 6 in the main text). During this time, the vaccination campaign was extended to individuals aged 65 years and older, as well as those aged 45–64 years with high-risk health conditions[13].

Examining the two scenarios for groups of individuals aged over 50 years, we observe that the combined effect of reduced susceptibility and changes in contact patterns marginalized the role in transmission of this group of adults. This effect is reflected in the structure of the NGM and, particularly, in its rows, as illustrated in Supplementary Figure 12. In counterfactual Scenario 1 (Supplementary Figure 5a), we see how maintaining constant susceptibility would drive the contribution of age group $[50, 70)$ above the average elasticity value 0.11 and R_t above the critical threshold of 1 by June 2021 (wave 24). On the other hand, in Scenario 2 (Supplementary Figure 5b), a drop in susceptibility while keeping contact patterns constant would slightly reduce the elasticity of adults aged over 50 years (particularly the group $[50, 60)$) maintaining R_t below 1.

23/06/21 - 03/09/21, survey waves 25 to 30

We focus on wave 25 (23 June 2021), when the Delta variant became the dominant strain in Belgium, and the vaccination campaign extended to all adults and adolescents in the age group $[12, 18)$. During this period, the age groups $[12, 18)$ and $[30, 40)$ showed the highest cumulative elasticity $\tilde{e}_j$ (see Figure 4 in the main text). We observe the evolution of the elasticity of groups in the age interval $[12, 40)$ under the same two scenarios until early September 2021 (wave 30), when the vaccination campaign had reached

70% coverage (two doses) and continued among adolescents in the $[12,18)$ age group, which recorded a minimum number of susceptibles at wave 30.

Supplementary Figure 6a illustrates that the variation in susceptibility among individuals aged 18 to 40 years helped limit their role in transmission, though not sufficiently to keep their elasticity below average (dashed lines). Supplementary Figure 6b shows that maintaining the contact levels recorded at wave 25 preserves the hierarchy of proportional contributions to R_t among these age groups. In both scenarios, the observed R_t remains notably lower than the hypothetical R_t (indicated by red asterisks), highlighting the combined importance of reduced contact activity and decreased susceptibility in determining R_t

To further investigate the interdependencies between these groups' roles in transmission, we examine the elasticity gradients presented in Supplementary Figure 7. By analyzing how variations in the number of secondary infections generated by individuals of age group m affect the cumulative elasticity indices By analyzing how variations in the number of secondary infections each infectious individual of age m is expected to generate, affect the cumulative elasticity indices(i.e., the sum of the gradient components in Equation (13)), we observe that the contributions of the $[12,18)$ and $[30,40)$ groups are strongly offset by variations in other age groups. In contrast, the $[18,30)$ group exhibits less sensitivity to such variations. This suggests that variations in social contacts, infectiousness, and susceptibility of highly interactive groups tend to reduce the transmission contribution of the $[12,18)$ and $[30,40)$ groups (which were already high at wave 25) while enhancing the role of the $[18,30)$ group, which has room for growth. We recall here that elasticity indices, as such, sum to 1.

The above scenarios indicate that changes in contact patterns significantly impact the age-specific proportional contribution at each wave. Susceptibility fluctuations appear to have a delayed effect and are less noticeable. Due to the consistent variation in social contacts reported in successive waves of the CoMix survey, along with significant fluctuations in susceptibles since April 2021, multiple factors contribute to redefining transmission hierarchies. This occurs regardless of keeping n NGM entries unchanged in Scenario 1 and $2n-1$ in Scenario 2, as the remaining entries vary. This, in turn, shifts the elasticity of each group, which can be explored using the formula (13).

Role of children during September 2021

We briefly focus on the NGM perturbation analysis at wave 31 (19 September 2021), where the elasticity measures highlight the predominant role in virus transmission of children under 12 years of age, with an $R_t = 1.05$. We aim to elucidate the effect of a hypothetical decrease in susceptibility in the age groups $[0,6)$ and $[6,12)$ on both R_t and the elasticity pattern.

Supplementary Figure 8a shows the linear approximation of the variation in R_t, calculated using sensitivity to changes in the number of susceptibles in different age groups, one by one. The bars represent the variation in R_t corresponding to a decrease in susceptible individuals in age group i of $\Delta S_i = -10^5$ and are obtained using formula (7). Such a variation in the $[6,12)$ group (corresponding to 14% of the pediatric population in that age) would be sufficient to push R_t just below the critical threshold of 1 (0.98). If this variation occurred in both groups under 12 years old, R_t would drop further to 0.95 (Supplementary Figure 8b).

Interestingly, despite high daily contacts (Supplementary Figure 9a) and a high infective value (Supplementary Figure 15), the $[12,18)$ group is a marginal contributor to transmission. This is due to a low sensitivity index, summarizing the effect of an extremely assortative contact pattern and low susceptibility (and infectiousness, see Supplementary Table 3). As a final counterfactual exercise, we modify the NGM of wave 31 by assuming a decrease in susceptibility in the under-12 groups and setting the susceptibility curve for $[12,18)$ at 90% of the level relative to wave 26 (7 July 2021, when the vaccination campaign extended to this group), compared to 60% recorded at wave 31. As shown in Supplementary Figure 9b, this combination of susceptibility would boost the proportional contribution of $[12,18)$ above the average.

NGM columns and rows

We recall the interpretation of the generic NGM entry k_{ij} as the expected number of secondary infections of age i generated by an infected individual of age j while infectious and briefly discuss the insights we can gain from Supplementary Figures 11 and 12. Supplementary Figure 11 clearly shows that the impact of new infections caused by an individual over 50 years old on the spread of the virus is minimal, especially after the Easter break and the rollout of vaccinations. If we add up the NGM columns corresponding to these age groups, the total never exceeds the threshold of 1. Using Equation (15), we conclude

that these groups lack the ability to sustain a pandemic outbreak due to their low force of infection. However, this does not apply to children and adults under 50 years old, as their NGM columns frequently exceed 1, indicating a potential contribution to an outbreak. Simultaneously, Supplementary Figure 12, through Equation (14), highlights that new infections among adults under 50 are particularly influential in sustaining the pandemic. In fact, the NGM rows sum measure the expected number of new infections in a specific group due to the interaction with an index case in each of the age groups, at each generation. We can think of the sum of row j as a measure of the overall susceptibility of age group j. By overall susceptibility, we mean the force of infection exerted by n index cases (one for each of the n age groups considered) on that group (j), scaled by their susceptibility. This is consistent with what we observe for minors in Supplementary Figure 12, due to their relatively low susceptibility combined with their tendency to interact with peers of similar age primarily, marginally breached the critical R_t threshold of 1 during specific periods: January to March 2021, June 2021, and September to October 2021. A notable deviation from these patterns is seen in adults between 50 and 60 years old. This group exhibited a pronounced tendency both to transmit the virus and to get infected until mid-April 2021. Subsequently, the surge in vaccine coverage led to a swift decrease in their susceptibility, as detailed in the main text. However, this reduced susceptibility observed a rebound by the end of July 2021, potentially attributable to waning vaccine-induced immunity and the proliferation of newer, immune-evading viral strains.

Supplementary Figures

Conterfactual Scenarios

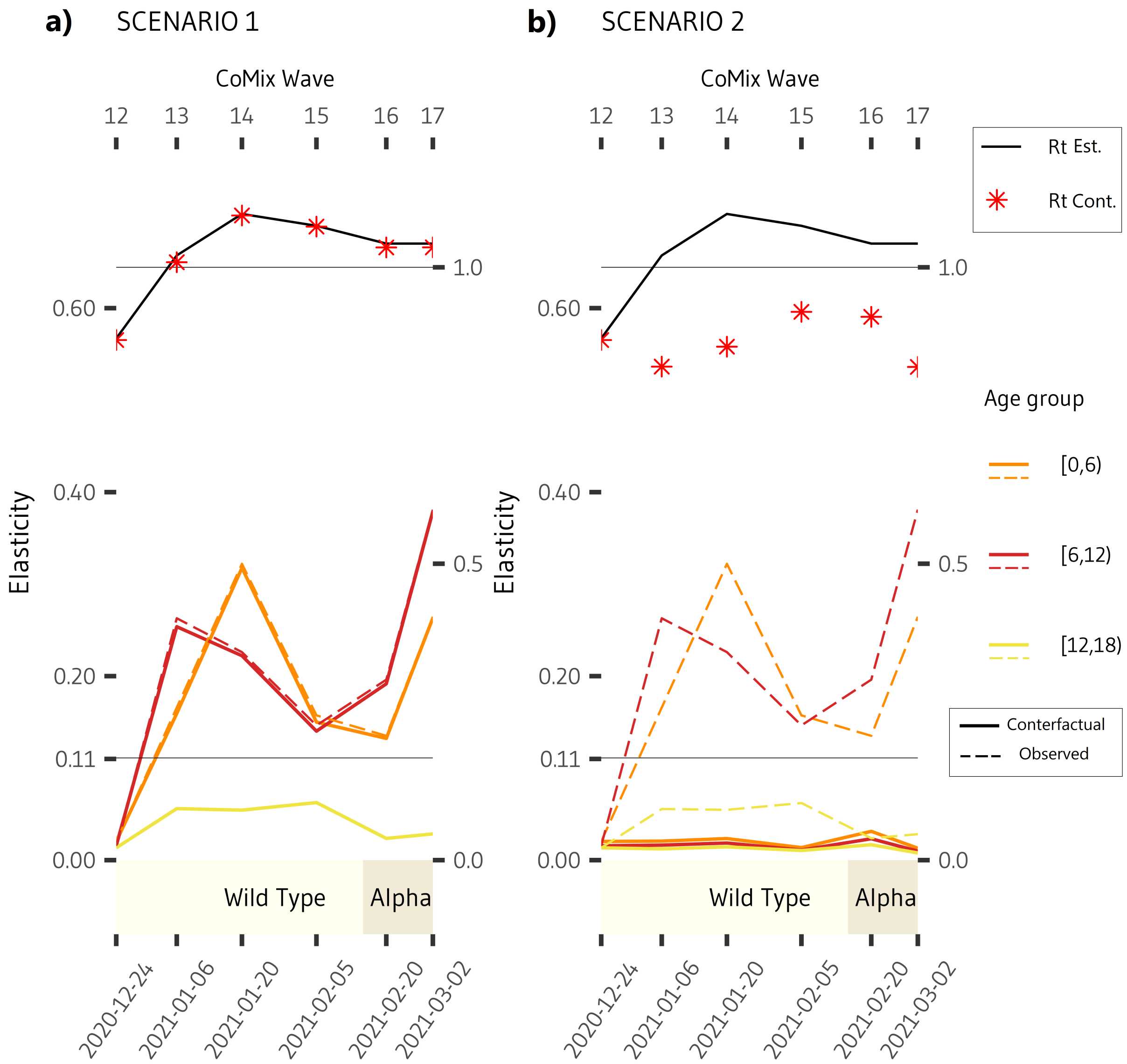

Figure 3: Impact of different scenarios on the elasticity pattern. (a) Scenario 1: Contacts of individuals under 18 years old vary according to observed data while maintaining a constant proportion of susceptibles at wave 12 levels. (b) Scenario 2: Contact levels are held constant at wave 12 levels. Solid-coloured lines represent counterfactual age-specific elasticity patterns, while dashed-coloured lines depict observed patterns. The solid black line shows the observed effective reproduction number (R_t), with red asterisks indicating counterfactual R_t values. Horizontal grey lines mark the average elasticity level (0.11, left y-axis) and the $R_t = 1$ threshold (right y-axis). The coloured bar at the bottom shows VOCs exceeding 50% of sequenced cases.

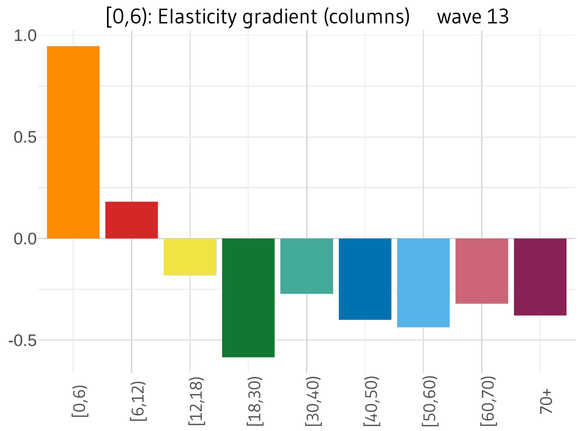

(a)

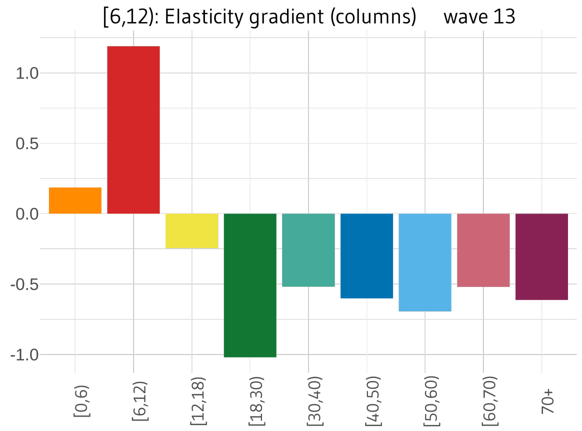

(b)

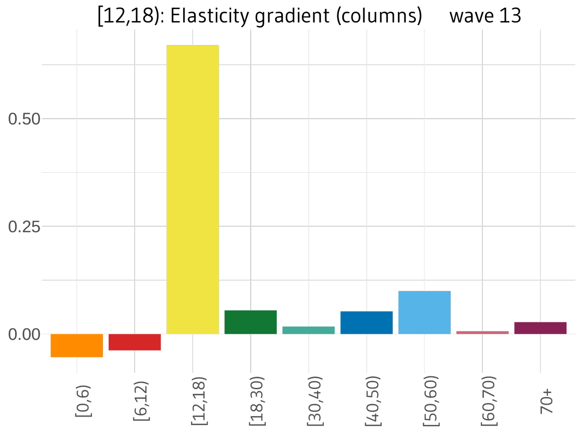

(c)

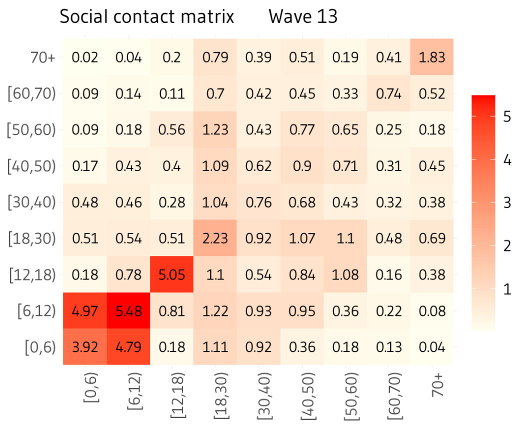

(d)

Figure 4: Elasticity gradient for age groups of minors during survey wave 13 (6 January 2021). Panels (a), (b), and (c) show $\sum_{l=1}^{n}\sum_{i=1}^{n}\frac{\partial e_{ij}}{\partial k_{lm}}$ for all age groups m with fixed j equal to $[0,6)$, $[6,12)$, and $[12,18)$, respectively. Panel (d) shows the social contact matrix for survey wave 13.

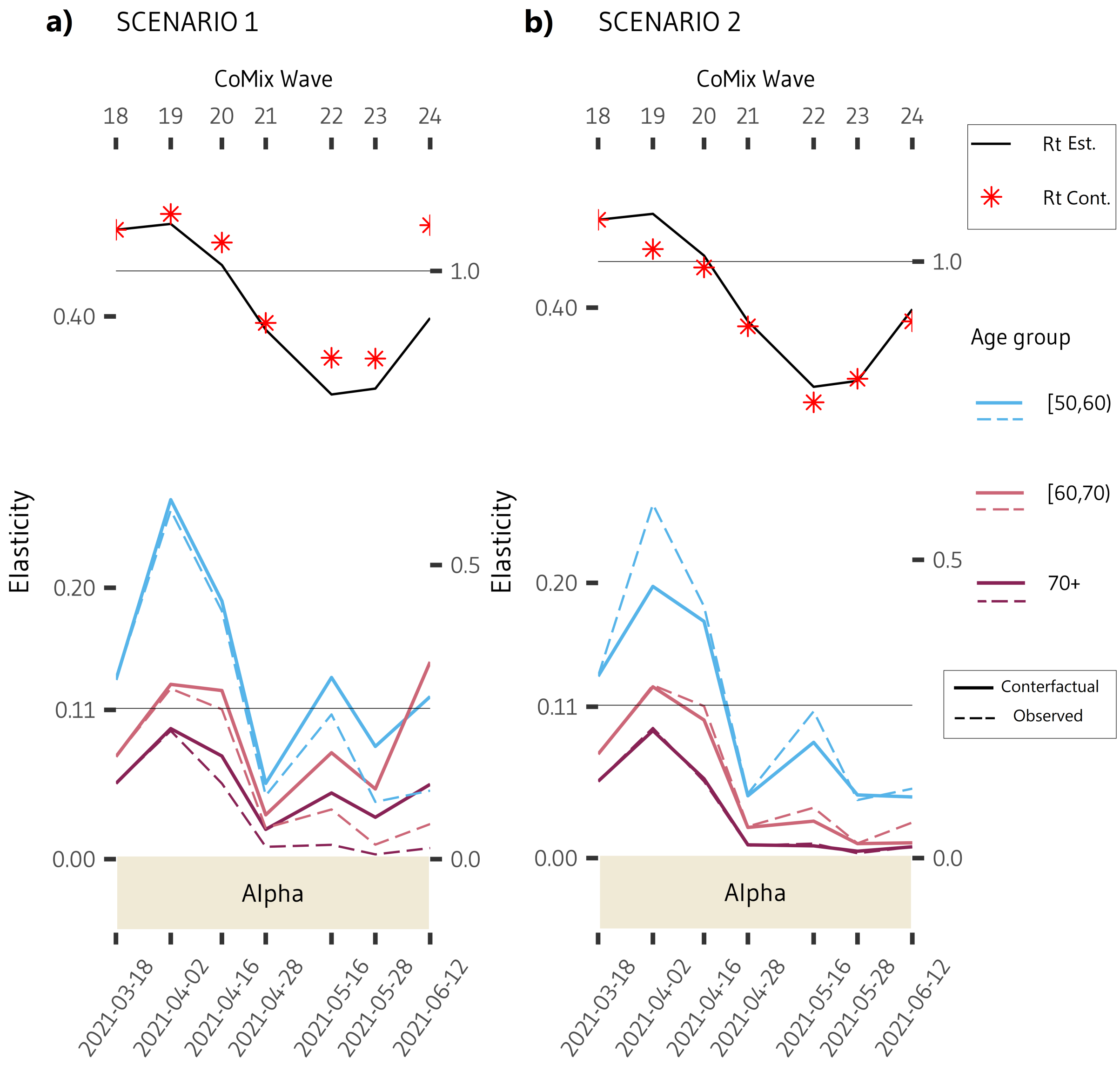

Figure 5: Impact of different scenarios on the elasticity pattern. (a) Scenario 1: Contacts of individuals under 18 years old vary according to observed data while maintaining a constant proportion of susceptibles at wave 18 levels. (b) Scenario 2: Contact levels are maintained at wave 18 levels. Solid-coloured lines represent counterfactual age-specific elasticity patterns, while dashed-coloured lines depict observed patterns. The solid black line shows the observed effective reproduction number (R_t), with red asterisks indicating counterfactual R_t values. Horizontal grey lines mark the average elasticity level (0.11, left y-axis) and the $R_t = 1$ threshold (right y-axis). The coloured bar at the bottom shows VOCs exceeding 50% of sequenced cases.

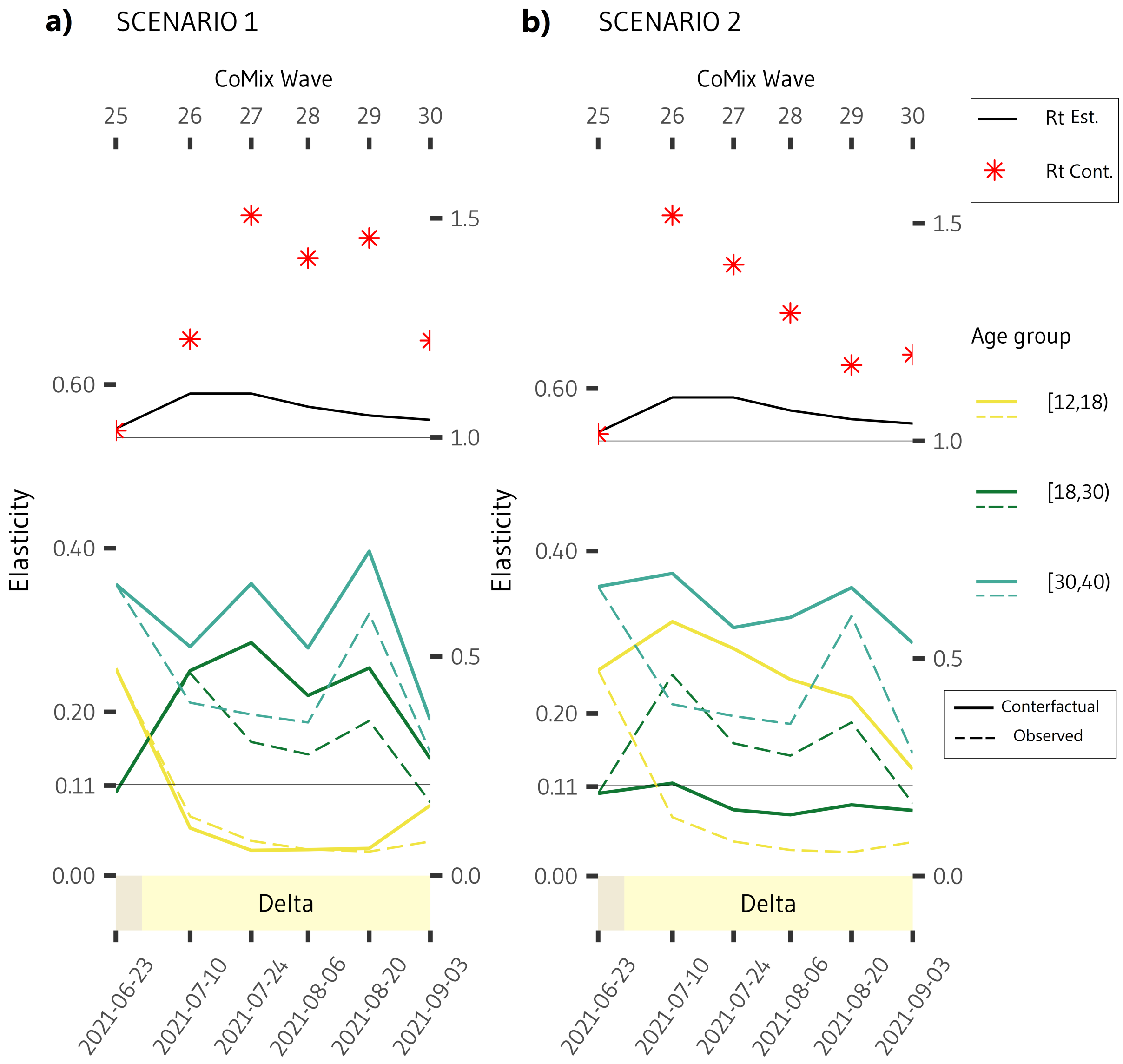

Figure 6: Impact of different scenarios on the elasticity pattern. (a) Scenario 1: Contacts of individuals under 25 years old vary according to observed data while maintaining a constant proportion of susceptibles at wave 25 levels. (b) Scenario 2: Contact levels are maintained at wave 25 levels. Solid-coloured lines represent counterfactual age-specific elasticity patterns, while dashed-coloured lines depict observed patterns. The solid black line shows the observed effective reproduction number (R_t), with red asterisks indicating counterfactual R_t values. Horizontal grey lines mark the average elasticity level (0.11, left y-axis) and the $R_t = 1$ threshold (right y-axis). The coloured bar at the bottom shows VOCs exceeding 50% of sequenced cases.

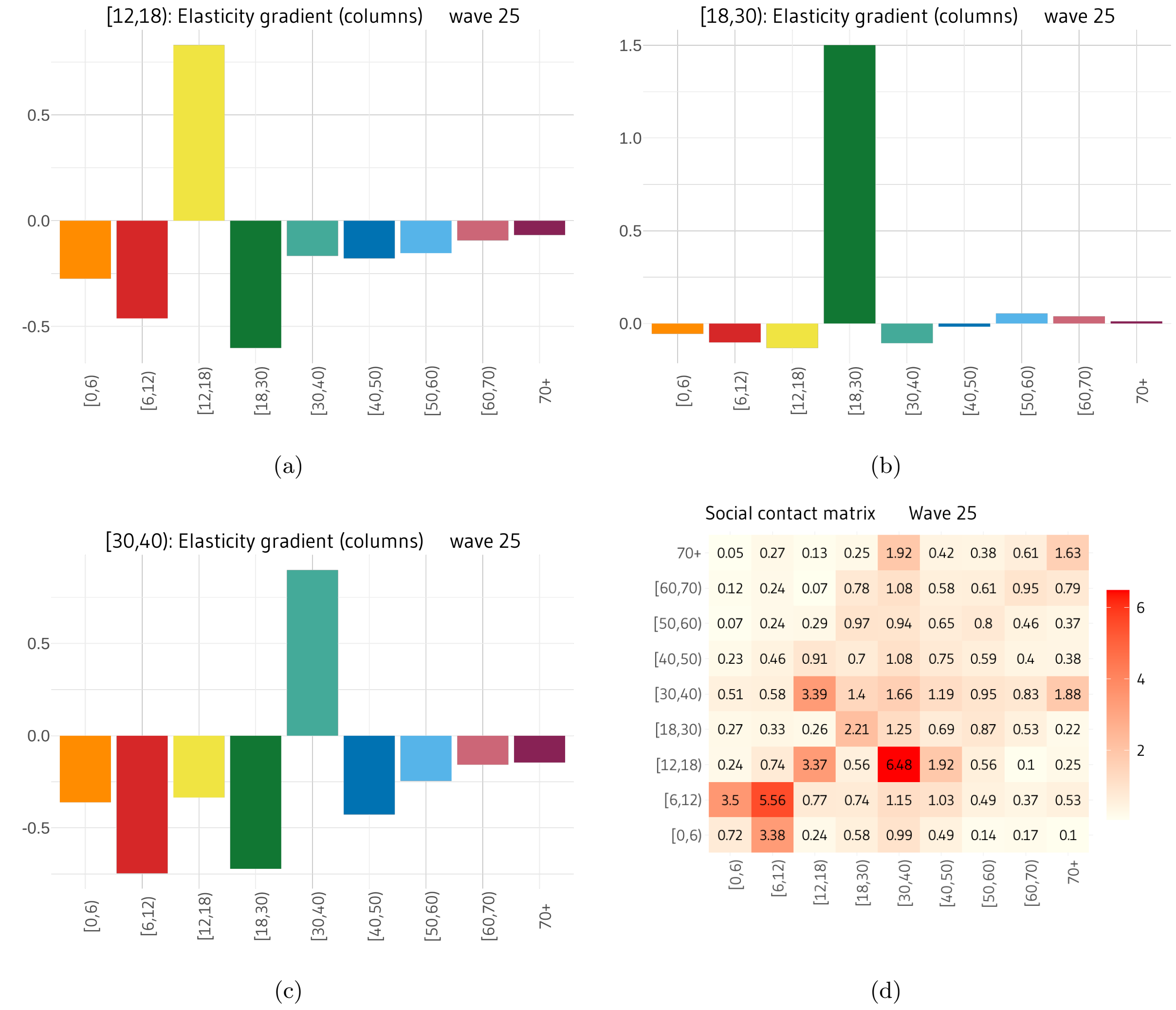

(a) (b)

(c) (d)

Figure 7: Elasticity gradient for age groups of individuals aged 12 to 40 years during survey wave 25 (23 June 2021). Panels (a), (b), and (c) show $\sum_{l=1}^{n}\sum_{i=1}^{n}\frac{\partial e_{ij}}{\partial k_{lm}}$ for all age groups m with fixed j equal to $[12, 18)$, $[18, 30)$, and $[30, 40)$, respectively. Panel (d) shows the social contact matrix for survey wave 25.

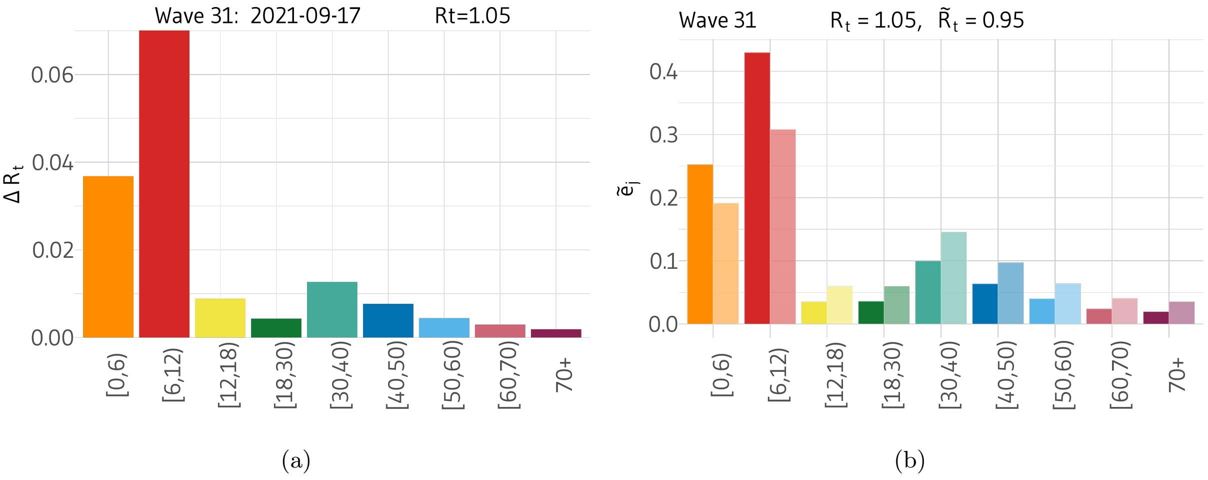

(a) (b)

Figure 8: Impact of decreased susceptibility on R_t during survey wave 31 (19 September 2021). Panel (a) shows the linear approximation of the R_t variation using the formula (7) for $\Delta S_i = -10^5$ in age group i. Panel (b) illustrates the scenario where this decrease is applied to the susceptible population under 12 years of age, reducing R_t to $\tilde{R}_t = 0.95$.

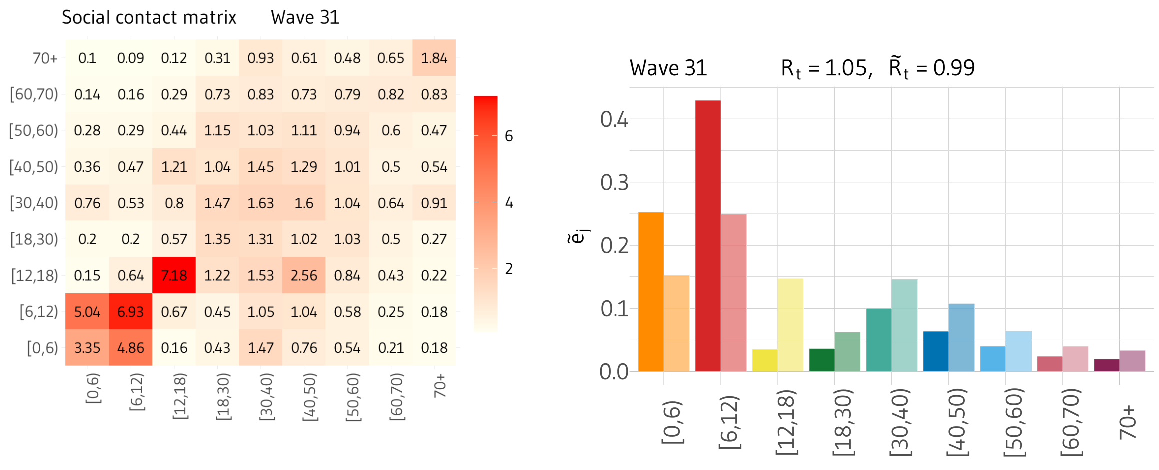

(a) (b) Scenario 2

Figure 9: Impact of adjusted susceptibility on [12, 18) age group's elasticity, survey wave 31 (19 September 2021). Panel (a) shows the contact matrix for survey wave 31. Panel (b) illustrates the scenario where the susceptible population under 12 years of age is decreased by $\Delta S_i = -10^5$, and the susceptible population in $[12, 18)$ is set to 90% of the levels calculated at wave 26 (7 July 2021).

Overall trends

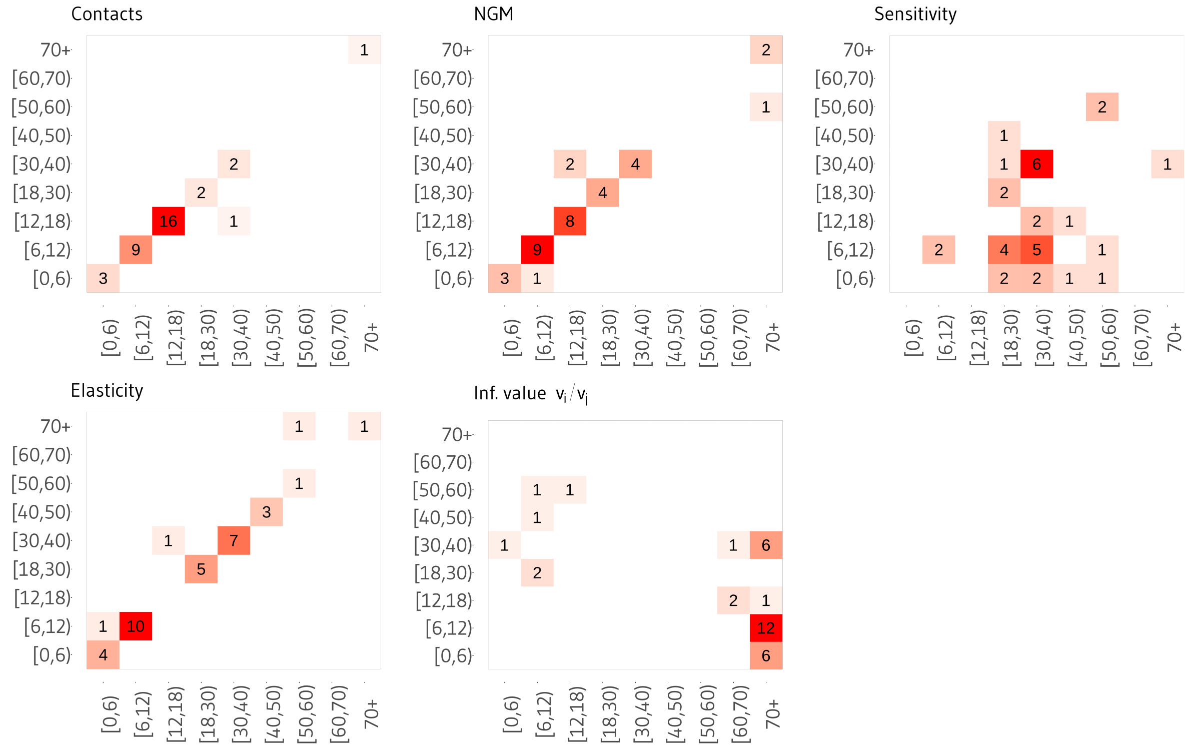

Figure 10: Maximal elements position. The heatmaps return the position of the maximal entry of several matrices utilized in the analysis at every observation point. The absolute frequency of a specific position (i, j) is reported in the cells.

Supplementary Figure 10 displays the frequency with which each matrix element (i, j) was the maximal element across 42 matrices, corresponding to our study period, respectively for the social contacts, next generation, sensitivity, elasticity and infective value ratio matrices. White cells correspond to entries that were never maximal. This visualization helps highlight which matrix elements consistently dominate under varying conditions. From the "Contacts" and "NGM" panels, we observe how the interactions

between individuals in age groups [6, 12) and [12, 18) were more often showing potential for transmission. The sensitivity indices refine this information by including the epidemiological feature related to the said interactions that govern the likelihood of transmission upon contact. Interestingly, in the "Sensitivity" and "Elasticity" panels, the intense, although assortative, mixing of the group [12, 18) never corresponds to the maximal sensitivity or elasticity element. On the other hand, the highest fluctuations in R_t are more often linked to the epidemiological parameters modelling the interactions between young adults in age group [18, 40) and children under the age of 12 ("Sensitivity" panel). The highest proportional contribution to R_t comes more often from the interactions between children in age group [6, 12) ("Elasticity" panel).

Other Figures

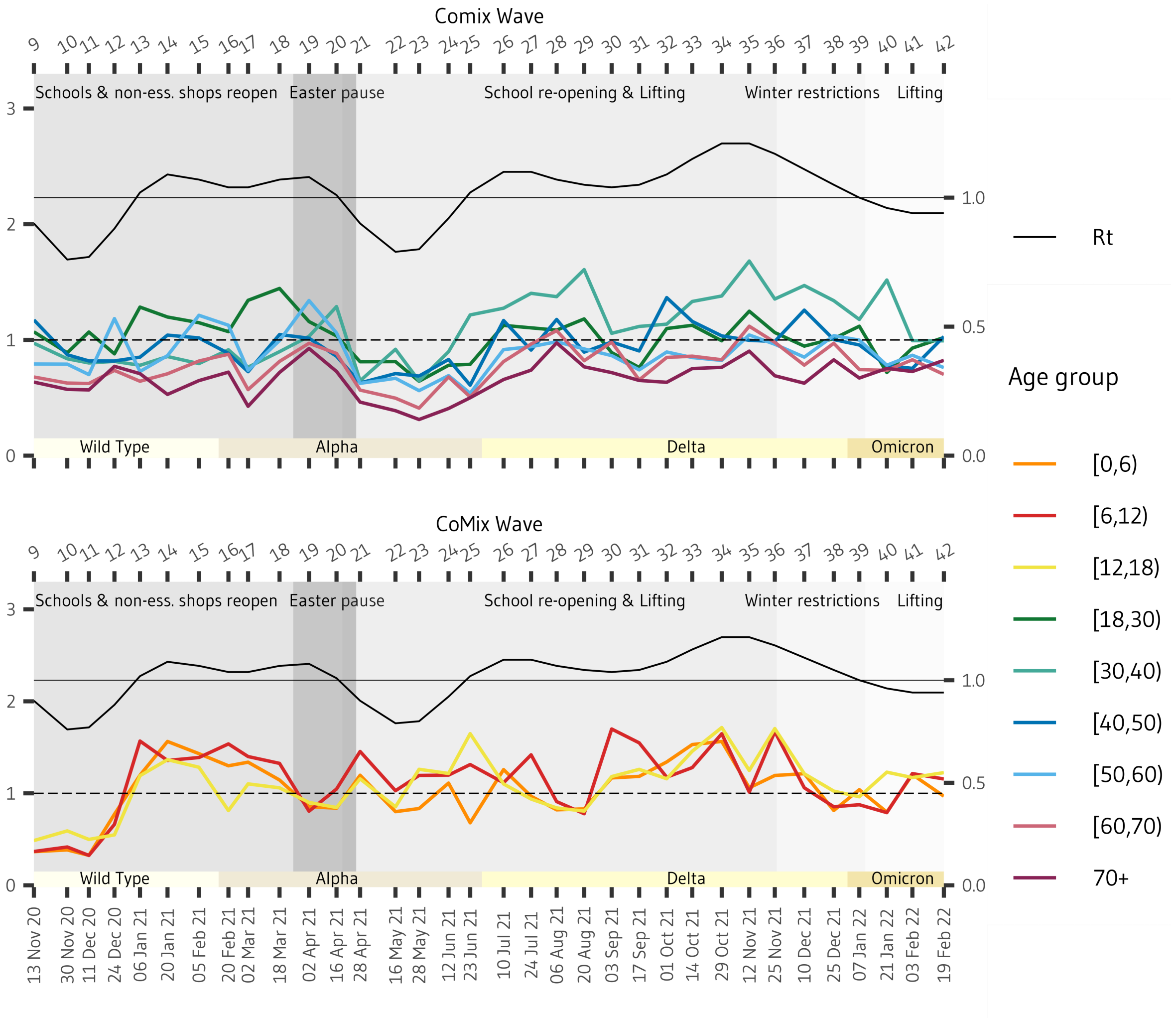

Figure 11: Evolution of the age-specific sum of the NGM column elements by age group. The sum of the elements in column j is interpreted as the expected number of secondary cases originating from an index case of age j while infectious, i.e. in one NGM iteration.

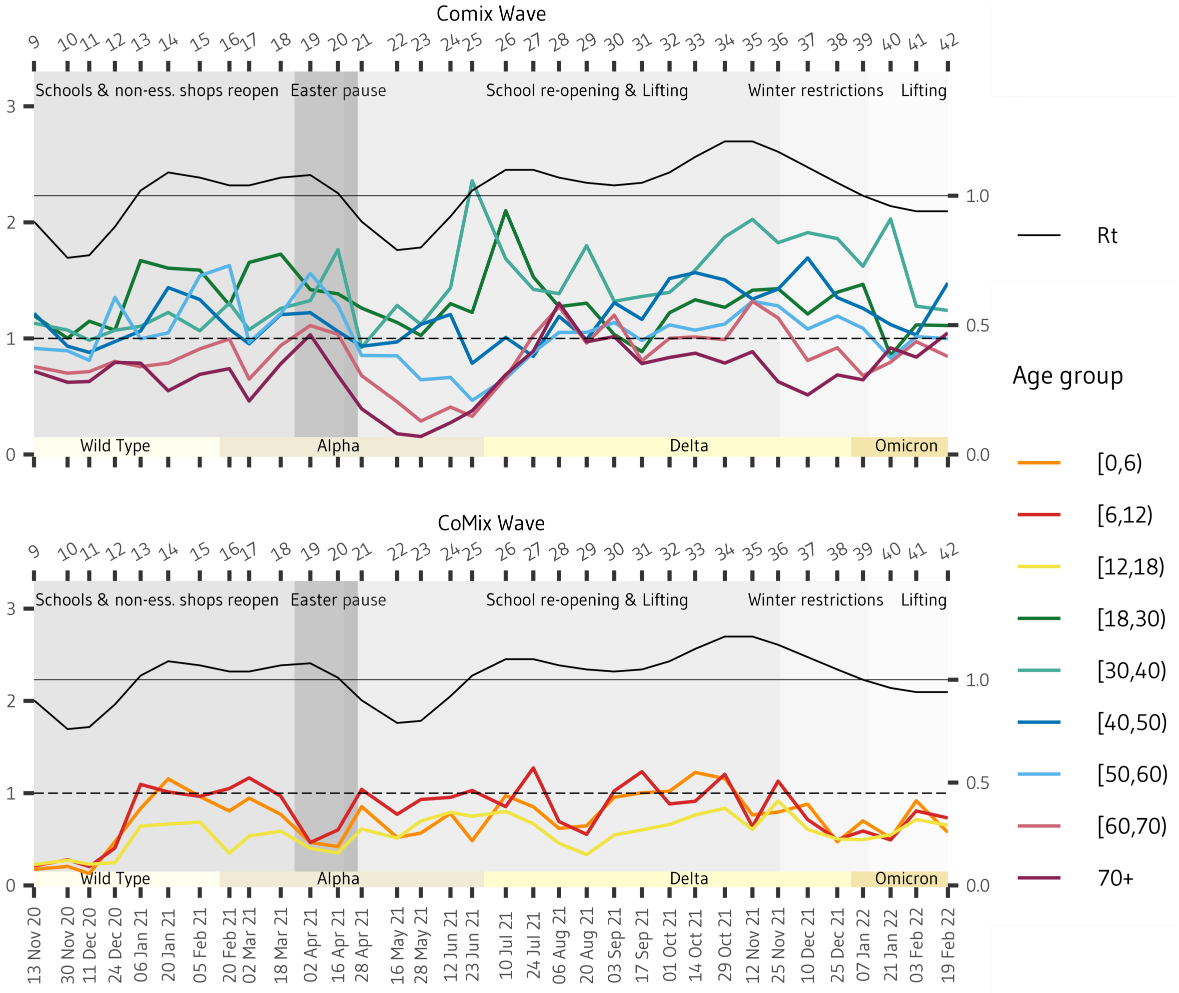

Figure 12: Evolution of the age-specific sum of the NGM row elements by age group. The sum of the elements in row j is interpreted as the expected number of secondary cases of age j that would result from interactions with an index case in each age group.

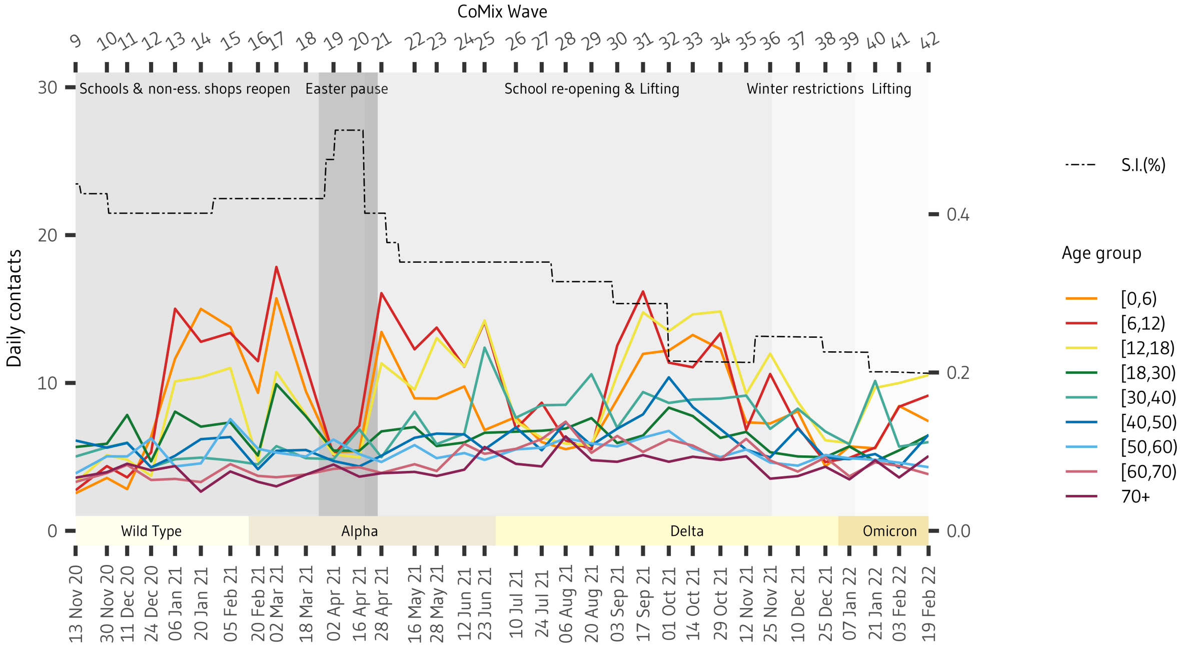

Figure 13: Reported daily contacts. The line chart shows the evolution of the average number of contacts reported by a participant to the CoMix survey at each survey wave and for each age group.

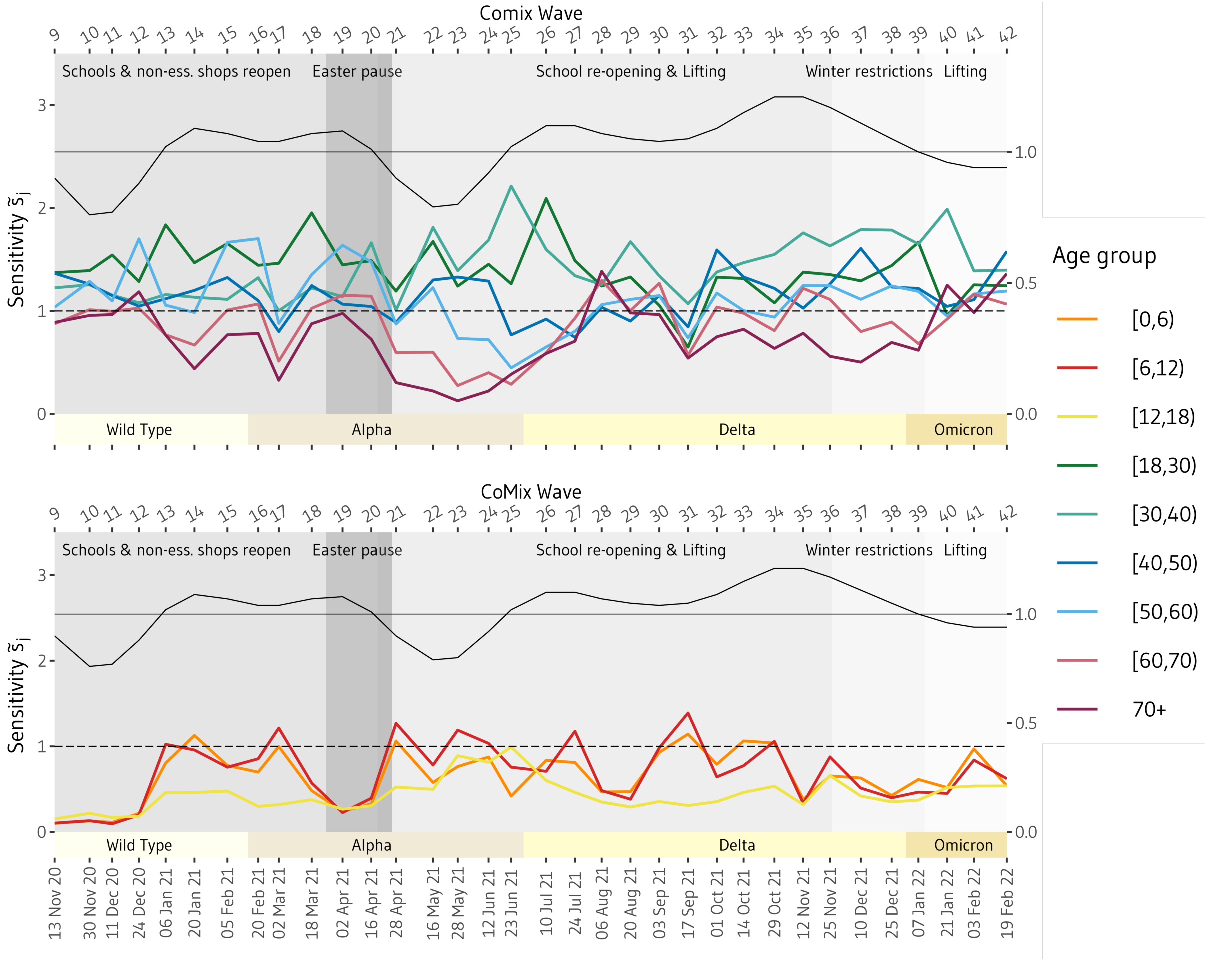

Figure 14: Age-specific sensitivity time series. On the x-axis, we report the sequence of CoMix waves; on the secondary x-axis(top), the corresponding calendar date. On the y-axis, we display the corresponding value of the cumulative sensitivity indices ($\tilde{s}_j$) relative to each age group. The dashed line indicates the average sensitivity value 1. In addition, the effective reproduction number (R_t) is plotted on a different scale indicated by the secondary y-axis (right), and a bar just above the main x-axis indicates the emerging variants of concern (VOCs) of SARS-CoV-2. The length of every coloured bar represents the duration during which the corresponding VOC was detected in more than 50% of sequenced SARS-CoV-2 cases.

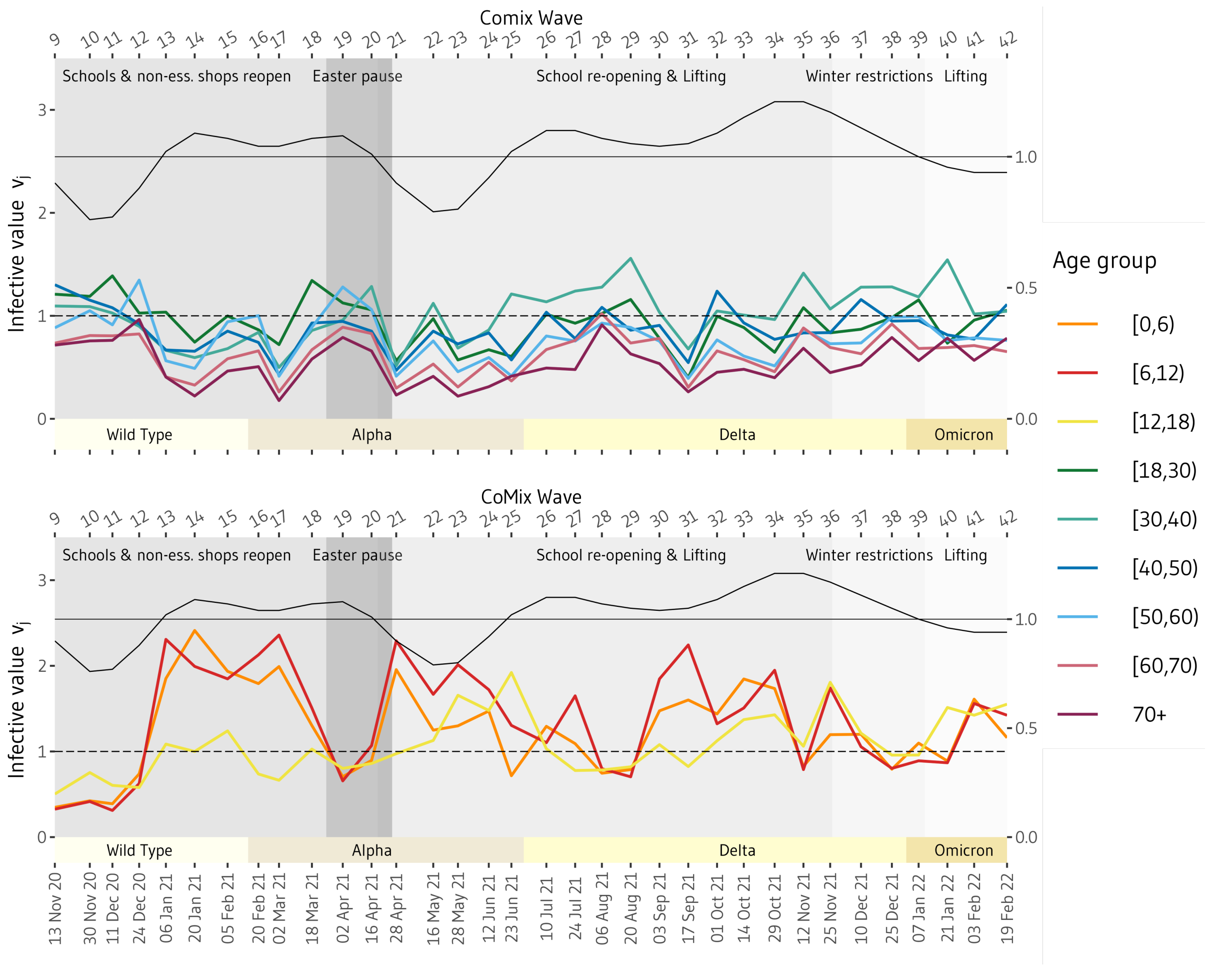

Figure 15: Age-specific infective value time series.we report the sequence of CoMix waves; on the secondary x-axis(top), the corresponding calendar date. On the y-axis, we display the corresponding infective values (v_j) relative to each age group. The dashed line indicates the average infective value 0.92. In addition, the effective reproduction number (R_t) is plotted on a different scale indicated by the secondary y-axis (right), and a bar just above the main x-axis indicates the emerging variants of concern (VOCs) of SARS-CoV-2. The length of every coloured bar represents the duration during which the corresponding VOC was detected in more than 50% of sequenced SARS-CoV-2 cases.

Supplementary Tables

wave	$\Delta_{R_{max}}$	Date	wave	$\Delta_{R_{max}}$	Date
9	0.62	13 Nov 20	22	0.52	16 May 21
10	0.25	30 Nov 20	23	0.42	28 May 21
11	0.20	11 Dec 20	24	0.36	12 Jun 21
12	0.55	24 Dec 20	25	1.19	23 Jun 21
13	0.95	06 Jan 21	26	1.19	10 Jul 21
14	0.62	20 Jan 21	27	0.61	24 Jul 21
15	0.50	05 Feb 21	28	0.66	06 Aug 21
16	0.74	20 Feb 21	29	0.41	20 Aug 21
17	0.67	02 Mar 21	30	0.75	03 Sep 21
18	0.46	18 Mar 21	31	0.60	17 Sep 21
19	0.61	02 Apr 21	32	0.57	01 Oct 21
20	0.45	16 Apr 21	33	0.51	14 Oct 21
21	0.86	28 Apr 21	34	0.46	29 Oct 21
22	0.52	16 May 21	35	0.68	12 Nov 21
23	0.42	28 May 21	36	0.65	25 Nov 21
24	0.36	12 Jun 21	37	0.59	10 Dec 21
25	1.19	23 Jun 21	38	0.55	25 Dec 21
26	1.19	10 Jul 21	39	0.43	07 Jan 22
27	0.61	24 Jul 21	40	0.59	21 Jan 22
28	0.66	06 Aug 21	41	0.69	03 Feb 22
29	0.41	20 Aug 21	42	0.55	19 Feb 22

Table 1: The table shows the values obtained for the maximal expected absolute variation in R_t (11) at each update of the NGM, corresponding to the ComMix survey waves 9 to 42. The average value calculated as the arithmetic mean over the observation period is $\Delta_{R_{max}} = 0.60$

wave	1	2	3	4	5	6	7	
Start	16 Apr '20	07 May '20	21 May '20	03 Jun '20	17 Jun '20	01 Jul '20	15 Jul '20	
End	28 Apr '20	14 May '20	28 May '20	11 Jun '20	24 Jun '20	08 Jul '20	22 Jul '20	
Center	19 Apr '20	10 May '20	22 May '20	05 Jun '20	18 Jun '20	03 Jul '20	18 Jul '20	
wave	8	9	10	11	12	13	14	
Start	29 Jul '20	11 Nov '20	26 Nov '20	09 Dec '20	22 Dec '20	05 Jan '21	19 Jan '21	
End	05 Aug '20	18 Nov '20	08 Dec '20	16 Dec '20	04 Jan '21	11 Jan '21	24 Jan '21	
Center	31 Jul '20	13 Nov '20	30 Nov '20	11 Dec '20	24 Dec '20	06 Jan '21	20 Jan '21	
wave	15	16	17	18	19	20	21	
Start	02 Feb '21	16 Feb '21	02 Mar '21	16 Mar '21	30 Mar '21	13 Apr '21	27 Apr '21	
End	07 Feb '21	23 Feb '21	04 Mar '21	24 Mar '21	06 Apr '21	19 Apr '21	03 May '21	
Center	05 Feb '21	20 Feb '21	02 Mar '21	18 Mar '21	02 Apr '21	16 Apr '21	28 Apr '21	
wave	22	23	24	25	26	27	28	
Start	12 May '21	25 May '21	09 Jun '21	22 Jun '21	06 Jul '21	20 Jul '21	03 Aug '21	
End	23 May '21	01 Jun '21	16 Jun '21	27 Jun '21	14 Jul '21	26 Jul '21	10 Aug '21	
Center	16 May '21	28 May '21	12 Jun '21	23 Jun '21	10 Jul '21	24 Jul '21	06 Aug '21	
wave	29	30	31	32	33	34	35	
Start	17 Aug '21	31 Aug '21	14 Sep '21	28 Sep '21	12 Oct '21	27 Oct '21	09 Nov '21	
End	23 Aug '21	07 Sep '21	20 Sep '21	04 Oct '21	17 Oct '21	03 Nov '21	15 Nov '21	
Center	20 Aug '21	03 Sep '21	17 Sep '21	01 Oct '21	14 Oct '21	29 Oct '21	12 Nov '21	
wave	36	37	38	39	40	41	42	43
Start	23 Nov '21	07 Dec '21	21 Dec '21	04 Jan '22	18 Jan '22	01 Feb '22	16 Feb '22	01 Mar '22
End	29 Nov '21	13 Dec '21	28 Dec '21	11 Jan '22	23 Jan '22	08 Feb '22	22 Feb '22	08 Mar '22
Center	25 Nov '21	10 Dec '21	25 Dec '21	07 Jan '22	21 Jan '22	03 Feb '22	19 Feb '22	04 Mar '22

Table 2: Overview of CoMix Survey waves: the above table provides the Start and End dates for each survey wave. The 'Center' day is determined by a weighted average of days elapsed since the start of the survey wave, with weights based on the number of questionnaires completed on a specific day. It is used to identify each wave throughout the analysis. We will focus on the period elapsing between wave 9 and 43, i.e. November 2020 - March 2022

Parameter	Description	Value (mean)	Source
τ_{inf}	infectivity ratio	0.51	[1, 4]
$\mathbf{p}$	age-specific probability of an asymptomatic COVID-19 case	$(0.94, 0.92, 0.90, 0.84, 0.61, 0.49, 0.21, 0.02, 0.02)$	[7]
$\mathbf{a}$	age-specific q-susceptibility	$(0.4, 0.39, 0.38, 0.79, 0.86, 0.8, 0.82, 0.88, 0.74)$	[10]
$\mathbf{h}$	age-specific q-infectiousness	$(0.54, 0.55, 0.56, 0.59, 0.7, 0.76, 0.9, 0.99, 0.99)$	[1]
c_{ij}	age-specific daily reported contact rate	-	[9]
γ	exposed removal rate	0.729	[1]
θ	pre-symptomatic removal rate	0.475	[1]
δ_1	asymptomatic removal rate	0.240	[1]
$\boldsymbol{\delta_2}$	age-specific recovery rate of mildly symptomatic cases	$(0.73, 0.74, 0.75, 0.74, 0.75, 0.74, 0.73, 0.72, 0.70)$	[1]
$\boldsymbol{\psi}$	age-specific rate of transition from mild to severe symptoms	$(0.021, 0.014, 0.006, 0.012, 0.010, 0.017, 0.022, 0.032, 0.050)$	[1]
$\boldsymbol{\omega}$	age-specific removal rate of severe symptoms cases	$(0.167, 0.131, 0.095, 0.099, 0.162, 0.338, 0.275, 0.343, 0.338)$	[1]

Table 3: Model parameters and their epidemiological meaning. The vector parameters components reflect the age structure corresponding to the age intervals $[0,6), [6,12), [12,18), [18,30), [30,40), [40,50), [50,60), [60,70), [70,\infty)$

**Vaccine type**	**Waning rate**	**Alpha**		**Delta**		**Omicron**	
		Infect.(1)	Hosp.(1)	Infect.(2)	Hosp.(3)	Infect.(2)	Hosp.(4)
Adeno: 1st dose	-	49%	76%	43%	76%(5)	18%	65%(6)
Adeno: 2nd dose	-	74%	86%	83%	95%	49%	81%(6)
mRNA: 1st dose	-	48%	83%	72%	79%(5)	32%	65%(5)
mRNA: 2nd dose	-	94%	95%	91%	99%	66%	81%
mRNA: booster dose	-	-	-	95%	99%(7)	67%	90%
Waned immunity after infection (*waning1*)	$\frac{1}{90\text{days}}$	63%	76%	63%	79%	9%	72%
Waned immunity after infection and vaccination (*waning2*)	$\frac{1}{90\text{days}}$	94%	95%	95%	99%	67%	90%
Waned immunity after two doses (*waning3*)	$\frac{1}{90\text{days}}$	63%	92%	63%	92%	9%	57%
Waned immunity after booster dose (*waning4*)	$\frac{1}{90\text{days}}$	-	-	89%	92%(7)	46%	81%(8)

Table 4: Vaccine- and infection-induced immunity levels against infection and hospital admissions by VOC and vaccine schedule, and waning compartments. (1) Lopez Bernal et al.[20]; (2) Andrews et al. [2]; (3) Andrews et al. [3]; (4) CDC Report, 2022 [23]; (5) Assumed equal to 80% of two doses; (6) Assumed equal to mRNA; (7) Assumed equal after 2 mRNA doses; (8) Assumed 90% of mRNA booster dose.

References

[1] Steven Abrams et al. "Modelling the early phase of the Belgian COVID-19 epidemic using a stochastic compartmental model and studying its implied future trajectories". In: *Epidemics* 35 (2021), p. 100449.

[2] Nick Andrews et al. "Covid-19 vaccine effectiveness against the Omicron (B. 1.1. 529) variant". In: *New England Journal of Medicine* 386.16 (2022), pp. 1532–1546.

[3] Nick Andrews et al. "Duration of protection against mild and severe disease by Covid-19 vaccines". In: *New England Journal of Medicine* 386.4 (2022), pp. 340–350.

[4] Leonardo Angeli et al. "Who acquires infection from whom? A sensitivity analysis of transmission dynamics during the early phase of the COVID-19 pandemic in Belgium". In: *Journal of Theoretical Biology* 581 (2024), p. 111721. ISSN: 0022-5193. DOI: https://doi.org/10.1016/j.jtbi.2024.111721. URL: https://www.sciencedirect.com/science/article/pii/S002251932400002X.

[5] Hal Caswell. *Matrix population models.* Vol. 1. Sinauer Sunderland, MA, USA, 2000.

[6] Hal Caswell. *Sensitivity analysis: matrix methods in demography and ecology.* Springer Nature, 2019.

[7] Taylor Chin et al. "Contact surveys reveal heterogeneities in age-group contributions to SARS-CoV-2 dynamics in the United States". In: *medRxiv* (2021).

[8] Pietro Coletti et al. "A data-driven metapopulation model for the Belgian COVID-19 epidemic: assessing the impact of lockdown and exit strategies". en. In: *BMC Infectious Diseases* 21.1 (Dec. 2021), p. 503. ISSN: 1471-2334. DOI: 10.1186/s12879-021-06092-w. URL: https://bmcinfectdis.biomedcentral.com/articles/10.1186/s12879-021-06092-w (visited on 07/10/2023).

[9] Pietro Coletti et al. "CoMix: comparing mixing patterns in the Belgian population during and after lockdown". In: *Scientific reports* 10.1 (2020), pp. 1–10.

[10] Nicholas G Davies et al. "Age-dependent effects in the transmission and control of COVID-19 epidemics". In: *Nature medicine* 26.8 (2020), pp. 1205–1211.

[11] Odo Diekmann, Hans Heesterbeek, and Tom Britton. *Mathematical tools for understanding infectious disease dynamics*. Vol. 7. Princeton University Press, 2013.

[12] Odo Diekmann, JAP Heesterbeek, and Michael G Roberts. "The construction of next-generation matrices for compartmental epidemic models". In: *Journal of the Royal Society Interface* 7.47 (2010), pp. 873–885.

[13] Food Chain Safety FPS Public Health and Environment. *Info-coronavirus (BE)*. https://www.info-coronavirus.be/. 2020. URL: https://www.info-coronavirus.be/.

[14] Nicolas Franco et al. "Inferring age-specific differences in susceptibility to and infectiousness upon SARS-CoV-2 infection based on Belgian social contact data". In: *PLoS computational biology* 18.3 (2022), e1009965.

[15] Oswaldo Gressani et al. "EpiLPS: A fast and flexible Bayesian tool for estimation of the time-varying reproduction number". In: *PLoS computational biology* 18.10 (2022), e1010618.

[16] Leonhard Held et al. *Handbook of infectious disease data analysis*. CRC Press, 2019.

[17] KULeuven. *UZ Leuven, Laboratoriumgeneeskunde, Department of Microbiology,Immunology and Transplantation, KU Leuven*. https://www.uzleuven.be/nl/laboratoriumgeneeskunde/genomic-surveillance-sars-cov-2-belgium. 2022.

[18] Max SY Lau et al. "Characterizing superspreading events and age-specific infectiousness of SARS-CoV-2 transmission in Georgia, USA". In: *Proceedings of the National Academy of Sciences* 117.36 (2020), pp. 22430–22435.

[19] Neilshan Loedy et al. "Longitudinal social contact data analysis: insights from 2 years of data collection in Belgium during the COVID-19 pandemic". In: *BMC Public Health* 23.1 (July 2023), p. 1298. ISSN: 1471-2458. DOI: 10.1186/s12889-023-16193-7. URL: https://doi.org/10.1186/s12889-023-16193-7 (visited on 09/13/2023).

[20] Jamie Lopez Bernal et al. "Effectiveness of Covid-19 vaccines against the B. 1.617. 2 (Delta) variant". In: *New England Journal of Medicine* 385.7 (2021), pp. 585–594.

[21] Mark EJ Newman. "Assortative mixing in networks". In: *Physical review letters* 89.20 (2002), p. 208701.

[22] S Unnikrishna Pillai, Torsten Suel, and Seunghun Cha. "The Perron-Frobenius theorem: some of its applications". In: *IEEE Signal Processing Magazine* 22.2 (2005), pp. 62–75.

[23] Diya Surie. "Effectiveness of monovalent mRNA vaccines against COVID-19–associated hospitalization among immunocompetent adults during BA. 1/BA. 2 and BA. 4/BA. 5 predominant periods of SARS-coV-2 omicron variant in the United States—IVY network, 18 states, december 26, 2021–august 31, 2022". In: *MMWR. Morbidity and mortality weekly report* 71 (2022).

[24] Katherine A Twohig et al. "Hospital admission and emergency care attendance risk for SARS-CoV-2 delta (B. 1.617. 2) compared with alpha (B. 1.1. 7) variants of concern: a cohort study". In: *The Lancet Infectious Diseases* 22.1 (2022), pp. 35–42.

[25] Thang Van Hoang et al. "Close contact infection dynamics over time: insights from a second large-scale social contact survey in Flanders, Belgium, in 2010-2011". In: *BMC infectious diseases* 21.1 (2021), pp. 1–15.

[26] Kim Van Kerckhove et al. "The Impact of Illness on Social Networks: Implications for Transmission and Control of Influenza". In: *American Journal of Epidemiology* 178.11 (2013), pp. 1655–1662. ISSN: 1476-6256. DOI: 10.1093/aje/kwt196. URL: https://dx.doi.org/10.1093/aje/kwt196.

[27] Frederik Verelst et al. "SOCRATES-CoMix: a platform for timely and open-source contact mixing data during and in between COVID-19 surges and interventions in over 20 European countries". In: *BMC medicine* 19.1 (2021), pp. 1–7.

[28] Lander Willem et al. "The impact of contact tracing and household bubbles on deconfinement strategies for COVID-19". en. In: *Nature Communications* 12.1 (Mar. 2021). Number: 1 Publisher: Nature Publishing Group, p. 1524. ISSN: 2041-1723. DOI: 10.1038/s41467-021-21747-7. URL: https://www.nature.com/articles/s41467-021-21747-7 (visited on 09/10/2023).

[29] Lander Willem et al. “The impact of quality-adjusted life years on evaluating COVID-19 mitigation strategies: lessons from age-specific vaccination roll-out and variants of concern in Belgium (2020-2022)”. In: *BMC Public Health* 24.1 (2024), p. 1171.
