# Supplementary material for: Assessing the role of children in the COVID-19 pandemic in Belgium using perturbation analysis
Source: Nat Commun. 2025 Mar 5;16:2230. doi: 10.1038/s41467-025-57087-z (PMC11882900; doi:10.1038/s41467-025-57087-z)
Supplement: Supplementary file 2 — Reporting Summary [file 41467_2025_57087_MOESM2_ESM.pdf]

nature portfolio

Corresponding author(s): Leonardo Angeli

Last updated by author(s): Feb 4, 2025

Reporting Summary

Nature Portfolio wishes to improve the reproducibility of the work that we publish. This form provides structure for consistency and transparency in reporting. For further information on Nature Portfolio policies, see our Editorial Policies and the Editorial Policy Checklist.

Statistics

For all statistical analyses, confirm that the following items are present in the figure legend, table legend, main text, or Methods section.

n/a	Confirmed	
☐	☒	The exact sample size (*n*) for each experimental group/condition, given as a discrete number and unit of measurement
☒	☐	A statement on whether measurements were taken from distinct samples or whether the same sample was measured repeatedly
☒	☐	The statistical test(s) used AND whether they are one- or two-sided<br>*Only common tests should be described solely by name; describe more complex techniques in the Methods section.*
☒	☐	A description of all covariates tested
☐	☒	A description of any assumptions or corrections, such as tests of normality and adjustment for multiple comparisons
☐	☒	A full description of the statistical parameters including central tendency (e.g. means) or other basic estimates (e.g. regression coefficient) AND variation (e.g. standard deviation) or associated estimates of uncertainty (e.g. confidence intervals)
☒	☐	For null hypothesis testing, the test statistic (e.g. *F*, *t*, *r*) with confidence intervals, effect sizes, degrees of freedom and *P* value noted<br>*Give P values as exact values whenever suitable.*
☐	☒	For Bayesian analysis, information on the choice of priors and Markov chain Monte Carlo settings
☒	☐	For hierarchical and complex designs, identification of the appropriate level for tests and full reporting of outcomes
☒	☐	Estimates of effect sizes (e.g. Cohen's *d*, Pearson's *r*), indicating how they were calculated

Our web collection on statistics for biologists contains articles on many of the points above.

Software and code

Policy information about availability of computer code

Data collection	We developed computer code in R 4.3.0, fully available at the following repository BELCovAge_v1.0.0: https://doi.org/10.5281/zenodo.14777392
Data analysis	We developed computer code in R 4.3.0, fully available at the following repository BELCovAge_v1.0.0: https://doi.org/10.5281/zenodo.14777392

For manuscripts utilizing custom algorithms or software that are central to the research but not yet described in published literature, software must be made available to editors and reviewers. We strongly encourage code deposition in a community repository (e.g. GitHub). See the Nature Portfolio guidelines for submitting code & software for further information.

Data

Policy information about availability of data

All manuscripts must include a data availability statement. This statement should provide the following information, where applicable:

- Accession codes, unique identifiers, or web links for publicly available datasets
- A description of any restrictions on data availability
- For clinical datasets or third party data, please ensure that the statement adheres to our policy

The datasets analysed in the current study are available in the Zenodo-based repository (https://zenodo.org/records/10549953), as well as through the CoMix-Socrates App (https://socialcontactdata.org/tools/).

Research involving human participants, their data, or biological material

Policy information about studies with human participants or human data. See also policy information about sex, gender (identity/presentation), and sexual orientation and race, ethnicity and racism.

Reporting on sex and gender	Not applicable. Our study primarily employs a mathematical modeling approach to analyze longitudinal social contact data, which has been sourced from a previously conducted survey study detailed in the references. The necessary ethical approvals and participant consent procedures pertinent to the original data collection are fully documented within those cited sources.
Reporting on race, ethnicity, or other socially relevant groupings	Not applicable. Our study primarily employs a mathematical modeling approach to analyze longitudinal social contact data, which has been sourced from a previously conducted survey study detailed in the references. The necessary ethical approvals and participant consent procedures pertinent to the original data collection are fully documented within those cited sources.
Population characteristics	Not applicable (N/a). Our study primarily employs a mathematical modeling approach to analyze longitudinal social contact data, which has been sourced from a previously conducted survey study detailed in the references. The necessary ethical approvals and participant consent procedures pertinent to the original data collection are fully documented within those cited sources.
Recruitment	N/a. Our study primarily employs a mathematical modeling approach to analyze longitudinal social contact data, which has been sourced from a previously conducted survey study detailed in the references. The necessary ethical approvals and participant consent procedures pertinent to the original data collection are fully documented within those cited sources.
Ethics oversight	N/a. Our study primarily employs a mathematical modeling approach to analyze longitudinal social contact data, which has been sourced from a previously conducted survey study detailed in the references. The necessary ethical approvals and participant consent procedures pertinent to the original data collection are fully documented within those cited sources.

Note that full information on the approval of the study protocol must also be provided in the manuscript.

Field-specific reporting

Please select the one below that is the best fit for your research. If you are not sure, read the appropriate sections before making your selection.

☒ Life sciences ☐ Behavioural & social sciences ☐ Ecological, evolutionary & environmental sciences

For a reference copy of the document with all sections, see nature.com/documents/nr-reporting-summary-flat.pdf

Life sciences study design

All studies must disclose on these points even when the disclosure is negative.

Sample size	The study relies on 34 consecutive datasets from the CoMix study, which define the observation period and provide data on social contacts in Belgium. These datasets include 4,592 participants and 182,986 recorded contacts. To account for potential biases in data reporting and sample representativeness, we computed weighted contact matrices for each wave, incorporating the Belgian age distribution and the day the questionnaire was completed. Given that the adopted methodology was developed based on deterministic assumptions, deriving average values was sufficient for our analysis.
Data exclusions	No data were excluded from the analysis.
Replication	The replication of our results is facilitated through the provision of both the code and data, which are publicly accessible at the repositories specified.
Randomization	N/a. This study employs mathematical modeling techniques. Detailed descriptions of the methods and data used are provided in both the main manuscript and the Supplementary Material, ensuring comprehensive documentation and accessibility.
Blinding	N/a. This study employs mathematical modeling techniques. Detailed descriptions of the methods and data used are provided in both the main manuscript and the Supplementary Material, ensuring comprehensive documentation and accessibility.

Reporting for specific materials, systems and methods

We require information from authors about some types of materials, experimental systems and methods used in many studies. Here, indicate whether each material, system or method listed is relevant to your study. If you are not sure if a list item applies to your research, read the appropriate section before selecting a response.

Materials & experimental systems

n/a	Involved in the study
☒	☐ Antibodies
☒	☐ Eukaryotic cell lines
☒	☐ Palaeontology and archaeology
☒	☐ Animals and other organisms
☒	☐ Clinical data
☒	☐ Dual use research of concern
☒	☐ Plants

Methods

n/a	Involved in the study
☒	☐ ChIP-seq
☒	☐ Flow cytometry
☒	☐ MRI-based neuroimaging

Plants

Seed stocks	N/a.
Novel plant genotypes	N/a.
Authentication	N/a.
